# Discovering sensorimotor agency in cellular automata using diversity search

**DOI:** 10.1126/sciadv.adp0834

**Published:** 2025-10-31

**Authors:** Gautier Hamon, Mayalen Etcheverry, Bert Wang-Chak Chan, Clément Moulin-Frier, Pierre-Yves Oudeyer

**Affiliations:** ^1^ INRIA, University of Bordeaux, Talence 33405, France.; ^2^ Poietis, Pessac 33600, France.; ^3^ Google DeepMind, Tokyo, Japan.

## Abstract

The field of artificial life studies how life-like phenomena such as agency and self-regulation can self-organize in computer simulations. In cellular automata (CA), a key open question is whether it is possible to find environment rules that self-organize robust “individuals” from an initial state with no prior existence of things like “bodies,” “brain,” “perception,” or “action.” Here, we leverage recent advances in machine learning, combining algorithms for diversity search, curriculum learning, and gradient descent, to automate the search of such “individuals.” We show that this approach enables us to systematically find environmental conditions in CA leading to self-organization of basic forms of agency, i.e., localized structures that move around and react in a coherent and highly robust manner to external obstacles, maintain their integrity, and have strong capabilities to generalize to new environments. We discuss how this approach opens new perspectives in artificial intelligence and synthetic bioengineering.

## Teaser

AI algorithms for curiosity-driven exploration enable automatic discovery of robust sensorimotor agents in cellular automata.

## INTRODUCTION

Understanding what has led to the emergence of life, cognition, and natural agency as we observe in living organisms has been a central debate across many sectors of life sciences. Biological organisms are made of collections of cells that follow low-level distributed rules, yet they constitute a coherent unitary whole, displaying strong *individuality* [words in italics are defined in the glossary (Box 1)] and *self-maintenance* in their environment, what was described to be an *autopoietic system*. While a central concept in theoretical biology, the characterization of an autopoietic system and the understanding of the processes underlying its self-organization remain a live issue. Further demystifying how these processes give rise not only to *organic individuation* but also to sensorimotor and even *intersubjective agency* is at the center of the debate (*1*).

Box 1. **Glossary.**

Individuality: Ability of a self-organizing structure (subpart of the environment) to preserve and propagate some spatiotemporal unity (*26*).

Self-maintenance: Ability of a self-organizing structure to modify its interactions with the rest of the environment for maintaining its integrity.

Autopoietic system: Introduced by Maturana and Varela (*50*), the concept of autopoiesis refers to a system capable of producing and maintaining itself by creating its own parts.

Organic individuation: Regulation at the metabolic, transcriptional, and morphological level to maintain organic integrity (*1*).

Intersubjective agency: Active engagement in communicative interactions and structural coupling with other agents (*1*).

Curriculum learning: Family of mechanisms that adapt the distribution of training environments to the learner capabilities (*71*).

Destructive perturbation: A perturbation is said to be destructive if it fundamentally disrupts the entity’s organization leading to its disintegration (*28*).

The concept of agency is viewed in diverse ways across disciplines, encompassing biological, philosophical, computational, and mathematical perspectives. Across those disciplines, agency is also often considered at different scales (*2*). Sultan *et al.* (*3*) use the term biological agency to refer to “the capacity of a system to participate in its own persistence, maintenance, and function by regulating its own structures and activities in response to the conditions it encounters.” At this biological scale, agency is thus fundamentally related to autopoiesis but requires in addition the asymmetrical regulation of the coupling with the environment (*4*–*6*), i.e., a “behavioral functionality.” Di Paolo (*1*) coined the term “sensorimotor agency” to describe a form of agency rooted in self-sustaining networked relations between sensorimotor schemes. This form of agency highlights the central role of behavioral functionality characterized by its own distinct norms related to skillful and coherent action, beyond just biological survival.

The different forms agency could take suggest that it might be better conceived as a continuum rather than a binary property of a system. This nonbinary (continuous) view is formalized in the TAME (Technological Approach to Mind Everywhere) framework proposed by Levin (*7*), where the ability to pursue “goals” is considered as the core feature of being a “self” but encompasses beings beyond the familiar conventional, evolved, static model animals with brains [down to gene regulatory network; (*8*)]. This nonbinary view is also present in (*6*), contrasting metabolic agency in basic autonomous systems, where self-construction and environmental interaction are inseparable, and neural agency in more complex organisms that is dynamically decoupled (although still dependent) from the metabolic basis. Computational models are relevant tools to study these different forms of agency in a formal manner. At the metabolic scale, cellular automata (CA) have been proposed as minimal models where most functional features of living beings (hence, agency) can be defined [(*9*, *10*); see, however, (*11*) for a critic]. At the cognitive scale, Biehl and Virgo (*12*) relies on the framework of partially observable Markov decision process (POMDP), proposing that an “agent” is simply a system together with an interpretation of this system as a POMDP solution, requiring the formation of goals and beliefs. Abel *et al.* (*13*) relies on the framework of reinforcement learning (i.e., a family of algorithms able to solve POMDPs) to highlight the subjective nature of agency, arguing that any measurement of a system’s agency must be made relative to a reference frame (e.g., defining a boundary between what is considered to be internal versus external to the agent). The term agent is widely used in artificial intelligence (AI) in general to refer to any computational entity able to sense, act, and make decisions in an environment (*14*).

**Fig. 1.**
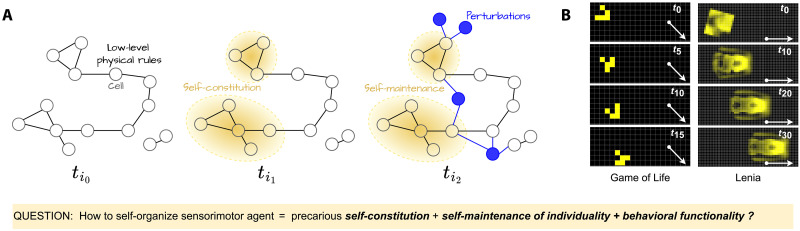
Overview of the scientific question. (**A**) The enactivist framework: ( $t_{i_{0}}$ ) In the beginning, there is only an environment made of low-level elements (cells) and physical laws (local rules). There is no prior notion of agency, no body, no sensor. ( $t_{i_{1}}$ ) Agents can come to existence through the coordination of the low-level elements (self-constitution of individuality). ( $t_{i_{2}}$ ) To maintain their integrity, agents must sense and react to perturbations using only local update rules (self-maintenance of individuality). (**B**) In CA models like the Game of Life and a more complex continuous extension called Lenia, it was shown that it is possible to self-organize so-called “gliders,” i.e., spatially localized patterns with directional movement. Directional movement (white arrows) and timesteps are displayed. (Question) In this work, following the enactivist modeling framework, we try to answer the following scientific question: Is it possible to find environments in which a subpart could self-organize and be called a “sensorimotor agent”? This would require the existence and emergence of glider-like structures that not only self-constitute and show motility, but are also robust to external perturbations and hence must develop some form of sensorimotor apparatus enabling them to make “decision” and “sense” at the macroscale through local interactions only.

Here, we focus on sensorimotor agency (*1*) and consider that a system exhibits such agency if it is able to (i) self-constitute its own morphology, (ii) self-maintain its own individuality, and (iii) exhibit behavioral functionality in interaction with its environment (Fig. 1). Our objective is to discover parameters of a complex dynamical system exhibiting such sensorimotor agency from a simulated substrate where it is originally absent. For this aim, we rely on the framework of continuous CA and propose a computational method for automating the search of patterns with sensorimotor agency, together with quantitative measures to characterize it.

Recent advances in biology and basal cognition suggest that many autopoietic systems that we find in nature, including plants and brainless animals, are robust sensorimotor agents capable of using a body for sensing opportunities, computing decisions, and acting in their environment (*15*). The pragmatic and complementary question to the debate, central in artificial life (ALife) and AI research, is whether we can engineer the necessary ingredients leading to the emergence of functional forms of life and sensorimotor agency in an artificial substrata in which initially there is literally no body (and, thus, no sensing, no acting, and no agent). Although there is already a large body of work that proposes to study the emergence of life and cognition in agents-as-they-could-be, it is generally either done by jumping over the biological processes that enable organisms to survive (the “mechanistic view,” as in, e.g., reinforcement learning, which considers a preexisting agent with predefined sensors and actuators) or inconclusive so far in showcasing higher-level forms of sensorimotor agency (the “enactivist view,” as in, e.g., artificial chemistry, which studies how some form of agency can emerge from low-level chemical reactions). Herein, after giving some background on the mechanistic and enactivist views on cognition and on their respective limitations, we suggest that modern tools from machine learning (ML) can help us bridge the gap between those two views. Whereas those tools have mainly been deployed within the mechanistic framework, we show that they can efficiently assist the discovery of environments that self-organize relatively advanced forms of sensorimotor agency whose existence and understanding are fundamental within the enactivist framework for supporting theories about the origins of life and cognition.

In the mechanistic view, one assumes the existence of agents that have well defined physical body and information processing brain allowing them to interact with the rest of the environment through predefined sensors and actuators. Robots, for instance, are referred to as embodied agents: Their individuality is clear, as they can easily be distinguished from the rest of the environment, and their self-maintenance is often not a problem, as their body does not change over time except for rare cases of real-world or artificially induced degradation. Hence, it is not questioned what makes an agent an agent or even what makes a body a body (*1*). Rather, a more central question is to understand how higher-level cognitive processes and sensorimotor adaptivity can arise in the agent through its interactions with the environment. A common methodology is the generation of a distribution of environments (tasks and rewards) and the use of learning approaches, such as deep reinforcement learning, to train the agent’s brain to master and generalize those tasks. Within that framework, it was shown that it is possible to engineer agents capable of repertoires of advanced sensorimotor skills such as precise locomotion (*16*), object manipulation (*17*), and tool use (*18*) and even capable of adapting the learned behaviors under unseen environmental conditions (*19*). They show that the use of *curriculum learning* is crucial to generate generally capable agents. However, the clear body/brain/environment distinction of the mechanistic framework bears little resemblance with the way information seems to be processed by biological systems. Notably, it goes against the concept of morphological computation (*20*), which argues that all physical processes of the body, not only electrical circuitry in the brain but also morphological growth and body reconfiguration, are integral parts of cognition and can achieve advanced forms of computation.

The enactive view on embodiment, however, is rooted in the bottom-up organizational principles of living organisms in the biological world. The modeling framework typically uses tools from dynamical and complex systems theory where an artificial system (the environment) is made of low-level elements of matter (called atoms, molecules, or cells) described by their inner states (e.g., energy level) and locally interacting via physics-like rules (flow of matter and energy within the elements) (Fig. 1A, $t_{i_{0}}$ ). There is no predefined notion of agent embodiment; instead, it is considered that the body of the agent must come to existence through the coordination of the low-level elements (Fig. 1A, $t_{i_{1}}$ ) and must operate under environmental perturbations and precarious conditions—the idea that bodies are constantly subjected to disruptions and breakdowns (Fig. 1A, $t_{i_{2}}$ ) (*1*). Hence, the self-constitution and self-maintenance of individuality are prior conditions for any agency to emerge, as they determine the agent’s own existence and survival (*1*). This shifts the problem of “building agents as-they-could-be” to a problem of engineering second-order emergence (*21*): how to design environments that can give rise to self-constituting agents that, coupled with the rest of environment, give rise to sensorimotor behaviors. Previous work has shown that the realization of autopoietic entities in computational media is possible (*22*–*25*). For instance, fully emergent structures showing spatial localization and movement have been discovered, such as the well-known gliders in the game of life up to richer life-like patterns in continuous models of CA (Fig. 1B). So far, however, two major challenges remain poorly addressed in the enactivist literature. First, autopoietic structures have so far mainly been discovered by human eye and as the result of time-consuming manual search, limiting their discovery and analysis. While some recent works, based on information theory tools, have proposed quantitative measures of individuality to facilitate their identification (*26*, *27*), their algorithmic implementation remains difficult in practice. Second, among the very few works that proposed a deeper analysis of the robustness capabilities of the discovered patterns (based on the enumeration of all possible perturbations that a structure can receive from its immediate environment) (*10*, *25*, *28*, *29*), findings suggest that glider-like structures typically remain quite fragile to external perturbations such as collision with other patterns (*10*).

In this work, we follow the enactivist framework and consider a class of continuous CA called Lenia (*30*, *31*) as our artificial “world.” In this system, we explore how local interactions between microentities can self-organize robust macrostructures (composed of many microentities) displaying coordinated and coherent sensorimotor functionality.

We present a method able to discover parameters of Lenia resulting in the formation of patterns robustly navigating a field of obstacles in the presence of internal and external perturbations. This type of sensorimotor functionality resembles the ones usually observed in the mechanistic framework, where the agent comes preequipped with a body, sensors, actuators, and a decision-making “brain.” In our enactivist simulations, however, the emergent macrostructure must operate this functionality only through the coordination of its microentities, all driven by the same local rules.

In particular, we show that modern tools from ML can help scientists explore the vast space of continuous CA dynamics, enabling to address the problem of engineering robust second-order emergence. Such exploration is challenging due to high dimensionality of the Lenia parameter space and the strongly nonlinear nature of its parameter-to-behavior mapping, where small variations of Lenia parameters can potentially result in drastic changes of the system behavior at the macro level (*32*, *33*). We propose a method based on curriculum learning, diversity search, and gradient descent, enabling us to efficiently shape the search process and to successfully navigate the chaotic outcome landscape of the high-dimensional Lenia system. In particular, we use a family of algorithmic processes called intrinsically motivated goal exploration process (IMGEP), an efficient form of diversity search algorithm (*34*). While mainly deployed in the fields of developmental robotics (*35*) and developmental AI to enable robots explore and map vast sensorimotor spaces (*36*, *37*), recent works have shown how IMGEP can also form useful scientific discovery assistants for revealing the range of possible behaviors in unfamiliar systems such as chemical oil-droplet systems (*38*), physical nonequilibrium systems (*39*), and models of continuous CA systems as the one considered here (*40*, *41*). At the difference of these previous works, we introduce two additional elements within the diversity search process: the use of gradient descent for local optimization and the use of stochastic perturbations within a curriculum of increasingly challenging and diverse target properties (hereafter called goals). With this method, we are able to find environmental rules leading to the emergence of patterns that self-constitute, self-maintain, and move forward under various obstacle configurations, i.e., autopoietic entities displaying robust forms of sensorimotor agency.

We then propose a battery of quantitative and qualitative tests, all formulated within the continuous CA paradigm, to further assess the robustness and generalization capabilities of the discovered self-organized patterns. The discovered sensorimotor agents also show strong robustness to several out-of-distribution perturbations ranging from perturbing the agent structure in various ways not seen during training (including by a collision with another agent) to changing the scale of the agent. Furthermore, when tested in a multi-entity initialization and despite having been trained alone, not only are the agents able to preserve their individuality, but they also show forms of coordinated interactions (attractiveness and reproduction), which could be interpreted as a primitive form of intersubjective communication (*10*). Those results illustrate the achievable generalization capabilities of artificial self-organizing agents, with respect to their mechanistic counterpart, opening interesting avenues for AI. At the same time, they provide interesting models about the way information might be processed by (brainless) biological agents to ensure robust maintenance of sensorimotor functions despite environmental and body perturbations (*42*).

### Study of sensorimotor agency in continuous CA models

CA are, in their classic form, a grid of “cells” $A=\left\{\;a_{x}\;\right\}$ that evolve through time $A^{t=1}\to \ldots \to A^{t=T}$ via the same local “physics-like” laws. More precisely, the cells sequentially update their state based on the states of their neighbors: $a_{x}^{t+1}=f\left[N\left(a_{x}^{t}\right)\right]$ , where x∈X is the position of the cell on the grid, $a_{x}$ is the state of the cell, and $N\left(a_{x}^{t}\right)$ is the neighborhood of the cell (including itself). The dynamic of the CA is thus entirely defined by the initialization $A^{t=1}$ (initial state of the cells in the grid) and the update rule *f* (how a cell updates based on its neighbors). However, predicting the system long-term behavior is a difficult challenge, even for simple rules, due to their potential chaotic dynamics (*43*).

In this work, we use Lenia, a class of continuous CA, which is a recently proposed generalization of Conway’s Game of Life (*30*, *31*). Previous works in Lenia have shown that there exist local update rules *f*, which can lead to the self-organization of long-term stable complex patterns that display interesting life-like behaviors (*30*, *31*, *41*). Those include forms of individuality (spatially localized organization), locomotion (directional movement), and even basic behavioral capabilities (change of direction in response to interaction with other patterns in the grid). However, in previous work, self-maintenance of those behaviors in discovered spatially localized patterns was typically quite fragile to external perturbations (for example, collision with other agents; movie S3), and properties of robustness and generalization were not specifically studied and tested: The possibility to self-organize robust self-maintaining agents was still an open question (and this applies to other CAs). Furthermore, these findings have so far relied on handmade exploration, which can be very hard and time-consuming as random rules rarely result in the emergence of localized patterns and even less moving ones (movie S2).

In this work, we propose to use AI techniques to automate experimentation and the exploration of Lenia, with minimal human intervention. More particularly, the automated experimentation aims to find local update rules *f* leading to the self-organization of stable (and, if possible, diverse) agents with sensorimotor capabilities. We also provide tests to assess the sensorimotor capabilities of the obtained patterns.

### The Lenia environment

Lenia is a class of continuous CA where each CA instance is defined by a set of parameters θ that conditions the CA rule $f_{\theta }$ . Once the parameters θ conditioning the update rule have been chosen, the system is a classical CA where the initial grid pattern $A^{t=1}$ is iteratively updated. In the multichannel version of Lenia (*31*), the system is composed of several communicating grids, which we call channels. Intuitively, we can see channels as the domain of existence of a certain type of cell. Each type of cell has its own physics: It has its own way to interact with other cells of its type (intrachannel influence) and also its own way to interact with cells of other types (cross-channel influence).

**Fig. 2.**
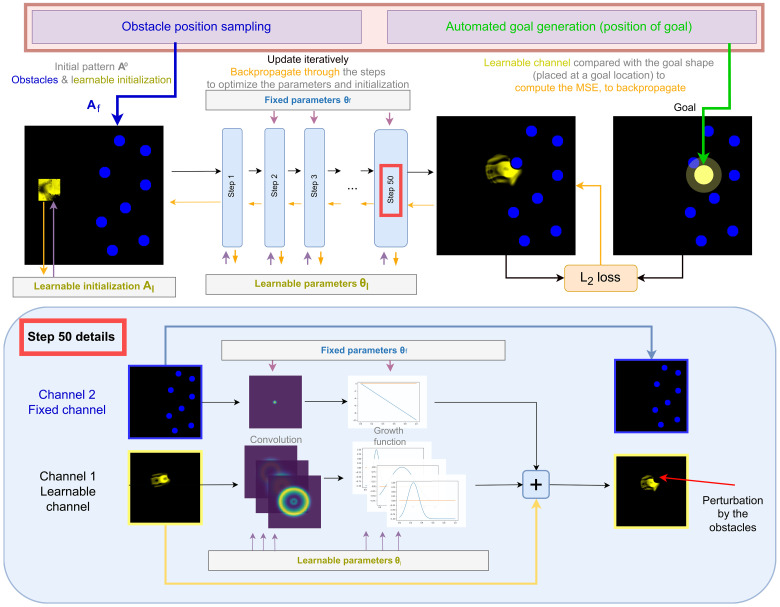
System overview. Top: Illustration of one experimental rollout with automated (i) generation of target goal (green), (ii) generation of environmental obstacles (blue), and (iii) optimization of learnable parameters toward goal (backpropagation shown in orange). The initial state is iteratively updated by the parameterized rule, and we then compute the goal conditioned loss from the last state of the rollout and propagate gradient across the steps to the learnable parameters and initialization. Bottom: Detailed view of a step in Lenia with obstacles. A convolution followed by a growth function is applied on each channel, resulting in a growth update, which is added to the current state of the learnable channel. Both the convolution and the nonlinear growth function in the learnable channel are parameterized (see section S11). MSE, mean square error.

In this work, we are interested in finding parameters ( θ, $A^{t=1}$ ) leading to the self-organization of moving agents robust to external perturbations from the environment. For this aim, we need to introduce perturbations in the system in a controlled systematic way, both for testing the robustness and as criteria during the search. However, due to the dynamical nature of the system, controlled perturbations over several steps in the CA system are often hard to introduce. To help solve this issue, we propose to take advantage of the multichannel version of Lenia and separate the low-level elements of the system in two types: The first “fixed” channel, which is hand-engineered, introduce elements that act as stable controlled obstacles (blue in Fig. 2); the second “learnable” channel, where parameters of the physic are learned, is where the agent has to emerge (yellow in Fig. 2). In practice, the environment parameters ( θ, $A^{t=1}$ ) are then separated in two. The first part, denoted ( $\theta_{f},A_{f}^{t=1}$ ) is a hand-engineered part where $\theta_{f}$ gives the rule on how obstacles block matter from going in, while $A_{f}^{t=1}$ gives the obstacle placement and shape. Details on how we implement obstacles as part of the CA rule can be found in Materials and Methods. The second part, however, denoted ( $\theta_{l},A_{l}^{t=1}$ ), is free: The method presented below enables us to learn these environment parameters so that agents with sensorimotor capabilities can self-organize.

What we are searching for is thus learnable parameters ( $\theta_{l},A_{l}^{t=1}$ ) that will induce a physic leading to the self-organization of sensorimotor agents that are able to move and survive in a grid where obstacles perturb their structure and therefore may break their integrity. Note that finding pattern with such capabilities is not trivial, for example, moving patterns found by hand in (*30*, *31*) (as the Lenia glider), which are stable without perturbations, often die from the collision with our engineered obstacles (movie S4). Note that in our system, if a sensorimotor agent is to emerge, the only way it can “sense” previously introduced obstacles is from the perturbations that the obstacles induce on its structure. Compared to the physical world, the pattern does not sense the obstacles by means of exchange of particles like photons or chemical molecules, as in vision or chemoreception, but more akin to direct touch as in haptic perception.

### Intrinsically motivated goal exploration process

Formally, a set of parameters $\left(A_{f},\theta_{f},A_{l},\theta_{l}\right)$ in Lenia maps to a certain sequence of states (trajectory **o**). This trajectory can then be mapped to a vector *R*(**o**), through a defined characterization function *R*. This vector provides a behavioral description of the trajectory, and the image of *R* represents the space of possible behaviors that can emerge in the system. As we will show below, randomly exploring the space of learnable parameters ( $A_{l},\theta_{l}$ ) is both costly in terms of experimentations and inefficient for finding robust sensorimotor behavior.

Thus, we propose to leverage an AI technique called IMGEP (*35*) to help exploring the space of behaviors. As this technique was originally developed to model curiosity-driven exploration in children (*44*), we call such a system a “curious automated discovery assistant.” The IMGEP technique relies on “goal-directed” search, which we leverage to drive the system toward the emergence of diverse target (sensorimotor) behaviors, called goals. More precisely, given a goal-sampling strategy *G*, IMGEP automatically samples target goals g ~ *G*, which are points in the behavioral space. For each goal g, the objective is then to optimize toward parameters $\left(\theta_{l},A_{l}\right)$ leading to a sequence of state that is mapped as closely as possible to this goal. To score the trajectory according to a goal, a loss function L(g,o) , which takes the trajectory and the goal as input, is used.

The behavioral descriptor *R* we choose here is the position of the center of mass at the last timestep of a simulation. The behavioral space then consists of all possible (*x*, *y*) coordinates in the grid. The objective for a given goal g=(x,y) is thus to find parameters $\left(A_{f},\theta_{f}\right)$ leading to the emergence of a spatially localized pattern attaining the goal position at the last timestep under several perturbation by obstacles. In this work, we choose to define the (goal-conditioned) loss as the mean square error between the state at the last timestep of the trajectory and a disk centered at the goal position. In addition to closeness to the goal position, the loss function we use incentivizes localization of the mass to prevent pattern explosion and collapse, which is a very common outcome of Lenia parameters. We then use gradient descent to optimize the learnable parameters ( $\theta_{l},A_{l}^{t=1}$ ) by backpropagating the loss through the steps and make progress toward the goal (Fig. 2).

Gradient descent optimization has already been successfully applied with CA (*45*) on learning CA parameters leading to the growth (and regrowth) of a target pattern (*46*) or texture (*47*), or enabling cellular collectives to perceive their large-scale structure (*48*), proving the effectiveness of such method (with some additional component for training for long-term stability) in complex chaotic self-organizing dynamic. However, in this work, we consider self-organizing autonomous patterns propagating in space while self-maintaining their own morphology. Such patterns are often called moving “solitons” (*31*), which are usually a fragile type of pattern in Lenia as moving in space in such a system means growing cells in the moving direction while destructing cells in the opposite direction. This equilibrium between growth and death is also challenged by the random perturbations we introduce in the system. This means that changes of parameters, because of the chaotic nature of the system (*33*), can easily break the equilibrium between growth and death of cells making the optimization harder.

To help with this difficult optimization landscape, we propose to introduce a “curriculum” for making small improvements iteratively. Curriculum learning has already been applied for optimizing CA rule with gradient descent as a solution for getting out of a trivial local optima in Variengien *et al.* (*49*). The curriculum also solves the technical gradient flowing problem, detailed in section S7.6.

**Fig. 3.**
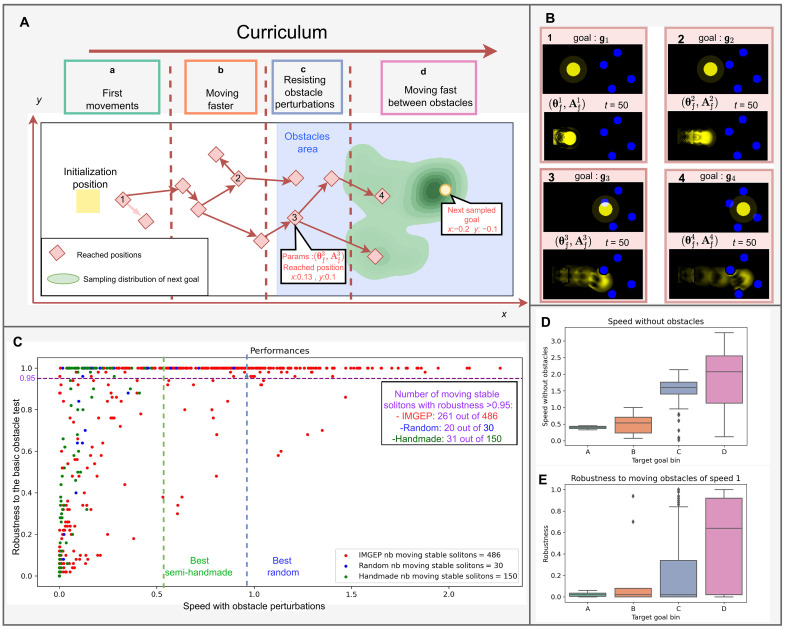
Curriculum and performances. (**A**) Schematic view of the curriculum. The curriculum iteratively samples goal positions (yellow disk), further in the grid, starting from very close to the initialization (a) to further away without obstacles (b) to further away in the obstacle area (c, then d). Arrow between reached positions (red square) represent that the parameters leading to a pattern attaining the tip of the arrow position was initialized before training by the parameters reaching the back of the arrow position. (**B**) Examples of patterns obtained along the curriculum as well as their associated goal. We observe patterns going further and further in the same amount of steps (50 steps) and for the latter dealing with obstacles in their way. To display the trajectory of the pattern in the learnable channel (yellow), we superposed the frames over all timesteps putting more transparency in earlier timesteps. (**C**) Performances in terms of robustness to the basic obstacle test and speed with obstacle perturbations of the moving stable solitons produced by IMGEP (red), random parameter search with the same computation as our method, i.e., 117,000 parameters tried in total (blue), and handmade solitons found in the original Lenia papers (green). (**D** and **E**) Distribution of the speed without obstacles perturbation (D) and robustness to moving obstacles (E) of moving stable solitons obtained by the IMGEP along the curriculum. Details on these metrics can be found in sections S8.3 and S8.5. We observe that the curriculum is translated in an improvement in the two presented quantities.

The intuitive idea behind our curriculum is to first learn rules leading to moving (spatially localized) patterns, which we train to go further and further (in the same amount of timesteps, hence faster), and, at some point, train them to go further while dealing with obstacles. To do so, the fixed environment $A_{f}^{t=1}$ we sample for training has a certain structure: The left half of the grid is free from obstacles, while the right part contains obstacles that will be randomly placed at every rollout (blue in Fig. 3A). The sampling strategy *G* we chose in the IMGEP also participates in the curriculum as it is biased to randomly sample goals that are a little bit further than previously attained positions. More information on the sampling strategy can be found in section S7.4. Putting target goals in the obstacle area means that during training, the emerging pattern will have to go to a specific location while its structure is perturbed by obstacles randomly placed. The gradient descent optimization will incentivize recovery from perturbation and to keep moving despite being damaged. In addition, the fact that the obstacles are randomly placed should incentivize generalization to different perturbations.

To sum up, the IMGEP iteratively (and automatically) generates increasingly difficult goals, in increasingly difficult and diverse environments, for which we will try to find, and optimize using gradient descent, learnable parameters ( $\theta_{l},A_{l}^{t=1}$ ) that will lead to the self-organization of patterns achieving these goals. For each goal (position), the optimization steps are done under several obstacle configurations $\left\{\;A_{f}\;\right\}$ to learn to resist to different perturbations. After each optimization, we then test the final obtained parameters on several obstacle configurations $\left\{\;A_{f}\;\right\}$ , which are sampled the same way as in the training steps, to assess the reached position. We store this (parameters, reached position) couple in history H to be able to use it as a starting point for subsequent goals. A more detailed description of the method can be found in Materials and Methods.

### Evaluation of the discovered patterns

In Introduction, we defined a sensorimotor agent as a system able to (a) self-constitute its own morphology, (b) self-maintain its own individuality, and (c) exhibit behavioral functionality in interaction with its environment. Following this definition, an instance of a sensorimotor agent in our proposed system would be a pattern able to form a stable soliton (fulfilling point a) and to navigate a field of obstacle (fulfilling point c) while resisting the perturbations they induce to its structure (fulfilling point b).

We propose to detect such patterns with the iterative method described as follows. First, an “empirical stable soliton filter” is used on the database of discoveries to discard parameters that do not lead to the self-organization of stable solitons in Lenia. More precisely, our filter implements several classifiers, inspired from ones proposed by Reinke *et al.* (*40*), to detect whether the emergent matter does not disintegrate (vanishes or explodes), forms a coherent entity (single soliton), and does so during a long-enough time window (longer than training). Then, we apply a moving filter that tells whether a soliton is moving (travels a minimum distance) or not (examples of discovered solitons that are considered not moving are shown in movie S19). Last, to assess the capabilities of the selected moving solitons to withstand perturbation by obstacles, we perform a basic obstacle test: testing their survival in the environment with different obstacle configurations similar to the ones seen during training. For this last test, we provide a distribution of perturbations (obstacle configurations) and measure “robustness” as the average performance over sampled perturbations, where performance is a binary success metric that determines whether the agent “survived” the perturbation or not. The robustness score is therefore a value between 0 and 1. As for the “survival” metric, we simply apply our stable soliton filter to detect whether the (perturbed) emergent entity is able to self-maintain despite the introduced perturbations (i.e., it is still a stable soliton at the end of the test). Note that this metric closely follows the definition of “cognitive domain” of an autopoietic system, which was introduced by Maturana and Varela (*50*) and later defined by Beer as the percentage of “nondestructive” perturbations, out of all possible perturbations, that the autopoietic system can tolerate (*28*). Because measuring the cognitive domain as such would require an exhaustive enumeration of all possible perturbations and all possible valid states that the entity can take, which are not tractable in the Lenia environment, we instead rely on a proxy metric and on a set of chosen empirical tests. In this work, we therefore consider a pattern as a sensorimotor agent if it (i) passes the soliton test, (ii) passes the moving test, and (iii) obtains a robustness score ≥0.95 in the basic obstacle test.

In addition to robustness, we also measure the performance of moving stable solitons in terms of speed with and without obstacles, especially as speed can be a measure of performance of motor capabilities (for example, for the biological agent to flee predators or chase preys) and as speed with obstacles is an interesting measure on how well the pattern deals with obstacles. Last, we also test the discovered sensorimotor agents to various generalization tests: running them through a battery of tests with several out-of-distribution perturbations that were not seen during training. In particular, we test the discovered sensorimotor agents in harder obstacle configurations, stochastic cell updates, changes of initialization, and changes of scale that were not experienced during training, computing robustness score for each of those perturbations in the same way as in test (iii) above. Refer to Materials and Methods and section S8 for more details on the evaluation procedure.

In addition, we provide the source code for reproducing the results at https://zenodo.org/records/10211741, enabling reproduction of all results, as well as an interactive web-demo at http://developmentalsystems.org/sensorimotor-lenia-companion/, where one can replay the discovered sensorimotor agents and test them to all sorts of freely drawn perturbations including custom obstacle shapes, addition and/or removal of mass, interactions with other agents in the grid, and control of environmental cues (attractive elements) in the Lenia grid. We argue that those quantitative and qualitative tests, which were all implemented within the continuous CA paradigm, can serve as a good baseline to evaluate the generalization capabilities (and hence the degree of agency) of autopoietic systems in enactivist research, akin to commonly deployed benchmarks in AI for evaluating mechanistic forms of agency (*19*).

## RESULTS

In this section, we analyze the discoveries made by the proposed approach (IMGEP) and compare it with two other exploration baselines: a “random search,” where parameters are sampled uniformly in the parameter space (same ranges as for the IMGEP, given in section S6.3), and a “handmade search,” where we collected the discoveries, made by semiautomatic search and expert selection, presented in the original Lenia papers (*30*, *31*). Each IMGEP experiment outputs 160 parameters but performs, in average, 11,700 Lenia rollouts, due to stochasticity in the method (see Materials and Methods). For IMGEP and random search, 10 independent repetitions are performed (where random search is given the same experimental budget of 11,700 rollouts per seed). Note that the comparison with handmade search, while interesting, is challenging in practice as it is the result of tedious search for which the total experimental budget is unknown, and which was conducted over some Lenia hyperparameters that are not all included in the automated search (e.g., various number of channels or kernels). Moreover, we use a slightly different parameterization of the rule to allow for differentiability (details in section S6.1). For the three baselines (IMGEP, random search, and handmade search), we filter the obtained parameters to select only the moving stable solitons (passing the stable soliton and moving test) and measure their speed and robustness to the basic obstacle test and generalizations tests, as described in the previous section.

### Individuality, locomotion, and sensorimotor capabilities

As illustrated in Fig. 3, the IMGEP search enables us to evolve patterns along a curriculum, which progressively leads to the emergence of individuality, locomotion, and sensorimotor capabilities. At first, the IMGEP samples goals (i.e., target positions) that are not too far from initialization (area a in Fig. 3A) and enabling us to find rules leading to the self-organization of spatially localized patterns, which start to move a little bit from initialization (as shown in Fig. 3B-1). Then, from these learned rules, the IMGEP samples further goals (area b in Fig. 3A), which lead to spatially localized patterns that move further in the grid in the same amount of time (Fig. 3B-2). At this point, some obtained parameters already lead to the self-organization of moving stable solitons, i.e., passing our empirical stable soliton test and moving tests (long-term stable solitons capable of moving while self-maintaining). Moving stable soliton patterns are in fact already not trivial to find through random search in the parameter space, as only 30 moving stable solitons were found through the 10 seeds of random search out of a total of 117,000 trials of parameters. The speed of the obtained moving stable solitons at this point is still limited as can be seen in Fig. 3D.

**Fig. 4.**
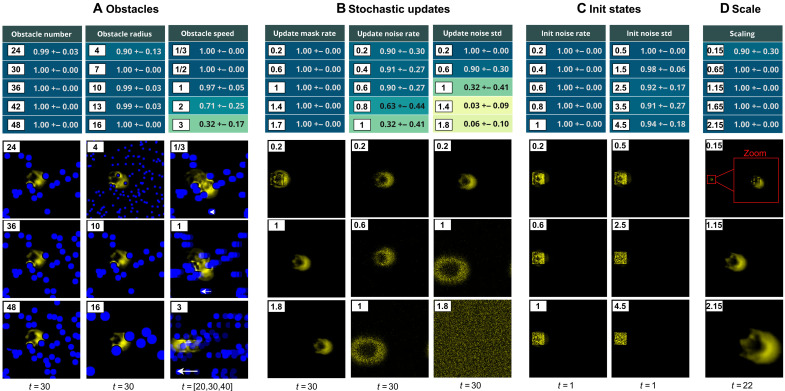
Quantitative tests of generalization of the discovered sensorimotor agents. We conduct a battery of quantitative tests, which we organize in nine families of parameterized perturbations that test for various (**A**) obstacle number, size, and speed; (**B**) rate of cell updates, as well as rate and magnitude of noise added to the updates; (**C**) rate and magnitude of noise added to the initial state (init); and (**D**) scaling factors. For each family, we test for five different parameter values, i.e., perturbation strength, resulting in a total of 9×5=45 tests. For each test, the performance of an agent is computed as the average score of survival over 10 random seeds. A score of 1 (dark blue) means that the agent survived all 10 tests, whereas a score of 0 (light yellow) means that the agent survived none of the tests. The table reports the mean and standard deviation (std) performances, over the 10 best agents discovered by our goal-directed curriculum, for all of the 45 tests (one table cell per test), where “best” is determined by the speed/robustness criteria introduced in Fig. 3C. Below each column, we show snapshots of system rollout at test time given the newly introduced perturbations. The shown snapshots are all taken from rollouts of the “best” agents, and from the first seed (out of the 10 tested random seeds). Timesteps are specified under the images; for instance, snapshots of the perturbations applied on the initial state are shown at *t* = 1.

The IMGEP pursues the curriculum, taking advantage of the previous learned parameters that already resulted in moving stable solitons, now sampling target goals that are even further away from the initial position, in the obstacle areas c and d in Fig. 3A, leading to moving stable solitons entering the obstacle area (as shown in Fig. 3B-3 and B-4). As expected, the parameters resulting from those goals have a higher robustness to obstacles as can be seen in Fig. 3E. We refer to section S3.1 for extra experiment with an ablation of the obstacle area during optimization showing that the increase of robustness is also due to the presence of obstacles in the optimization and not only to the distance of the target goal position to the initialization.

As expected, we observe that moving stable solitons trained with further goals move in average at faster speeds in the environment without obstacles (Fig. 3D). At the end of the curriculum loop, the obtained rules often lead to the self-organization of moving stable solitons that are able to navigate fast in an area with obstacles while still maintaining their integrity (Fig. 3B-4 and movie S1). In total, 9 of 10 seeds led to at least one sensorimotor agent, i.e., moving stable solitons with a measured robustness of ≥0.95 in our basic obstacle test. In practice, they are capable of changing direction and recovering in response to perturbations induced by the obstacles. This behavior is achieved only through the global coordination of identical low-level parts and, in particular, without having any central unit computing decision. Note, however, that the performance in terms of speed with obstacles varies from one seed to another (see table S1).

Over the 10 seeds, a great part of the obtained emerging moving stable solitons is sensorimotor agents. Over 10 seeds, 486 of the 1600 parameters (10 seeds × 160 parameters) led to moving stable solitons according to our empirical stable soliton and moving filter, from which 261 have a robustness to obstacles of ≥0.95.

As a comparison, out of the 117,000 parameters generated by the 10 seeds of random search, only 30 led to moving stable solitons from which 20 have a robustness to obstacles of >0.95. Our method surpasses random search in terms of speed with obstacles and robustness, as well as the total number of long-term stable moving solitons obtained, as can be seen in Fig. 3C (486 for IMGEP and 30 for random search in total over 10 seeds and with the same Lenia rollout budget). Random search is able to find some stable solitons (~1% of all its discoveries), but most of them are static compared to IMGEP, whose directed search fosters the emergence of moving stable solitons (movie S5).

Our method also results in moving stable solitons with better robustness and speed than the ones found in the original Lenia papers (Fig. 3C) (*30*, *31*). Ablation studies of the method can be found in the section S3, showing how curriculum, diversity search, and gradient descent are key ingredients in the method and are an efficient direction to search for sensorimotor behavior in self-organizing systems. We also provide the sequence of reached positions of a seed in section S2, displaying the curriculum and showing how diversity search can help find potential stepping stones.

### Generalization

**Fig. 5.**
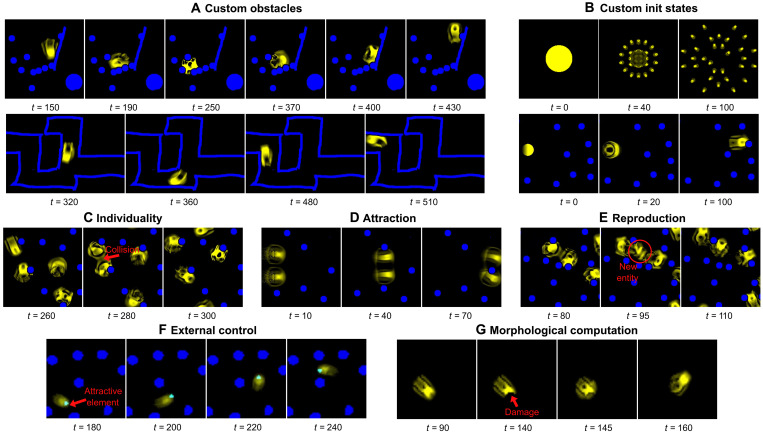
Qualitative tests of generalization of the discovered sensorimotor agents. We conduct a battery of qualitative tests, where we test the (best) discovered sensorimotor agents to all sorts of difficult perturbations including (**A**) freely drawn obstacles such as walls, mazes, or dead ends; (**B**) freely drawn initial states such as very big disks (resulting in the emergence of multiple entities) or small disks with gradient asymmetry; (**C** to **E**) introduction of other agents in the grid (resulting in the emergence of interagent interactions such as individuality maintenance, attraction, and reproduction); (**F**) the introduction of additional low-level elements that have an “attractive” effect on the sensorimotor agents (allowing an external user to guide the agent trajectory in the grid); and (**G**) custom mass removal (pixel erasing). Details of the resulting observed behaviors are provided in the text, with movies available on the companion website https://developmentalsystems.org/sensorimotor-lenia-companion/.

Biological organisms are able to maintain phenotypic stability in the face of diverse environmental perturbations arising from external stresses, intracellular noise, and even quite drastic changes during morphogenesis such as perturbations to the embryo structure (*51*) or to the substrate cellular size (*52*). It has long been recognized that robustness is an inherent property of all biological systems that has been strongly favored by evolution (*53*). In this section, we are interested to see whether similar robustness capabilities can be achieved by the artificial self-organizing patterns that have been discovered by our artificial evolution workflow (Figs. 4 and 5). To do so, we evaluate the generalization capabilities, over the proposed battery of tests, of the 10 best sensorimotor agents discovered by the IMGEP, random search, and handmade search variants, as well as on the moving stable solitons that have a speed within obstacles greater than one (91, all discovered by IMGEP). “Best” here is computed according to the speed-robustness criteria presented in Fig. 3C, i.e., the fastest with obstacle that also have a robustness in the basic obstacle test of >0.95. The performances are fully reported and compared in table S2. As we will see, the discovered sensorimotor agents showcase strong generalization capabilities under diverse conditions not experienced during the search.

We group the observed generalization capabilities into six categories: harder obstacle configurations (external stresses), stochastic cell updates (per-cell noise), changes of initialization, changes of scale (compute capacity variation), and interactions with other patterns in the grid (interagents regulation) as well as with human-controlled environmental cues (observer-agent regulation).

#### Harder obstacles

We first tested the sensorimotor agents’ generalization capabilities to a larger and more challenging set of obstacle configurations. The test set includes controlled configuration with varying number, size, and speed of obstacles (Fig. 4A), as well as human-drawn obstacles such as vertical walls and dead ends (Fig. 5A). Whereas some well-placed perturbations can lead to death or explosion, the discovered sensorimotor agents show strong robustness and generalization to most of the test set configurations. They showed quasiperfect survival to grids with up to 48 obstacles, to grids with small (but dense) or big (but sparser) obstacles, and to obstacles with moderate speed. High-speed obstacles, however, seem to challenge the sensorimotor agent’s survival (Fig. 4A), even though the IMGEP-discovered sensorimotor agents are still much more robust to moving obstacles than the ones discovered by random and handmade search (table S2 and movie S6). Those results suggest that, by training for fast-moving and obstacle-resisting behaviors, our goal-directed curriculum favored the self-organization of sensorimotor agents that are able to quickly recover from perturbations induced by the environment, even ones not seen during training. For instance, qualitative tests also showed that the discovered sensorimotor agents are able to successfully navigate forward while coming across tightly packed obstacles, walls of various inclinations, corners, dead ends, and even bullet-like types of obstacles (movie S10).

#### Stochastic updates

We then tested the generalization capabilities to asynchronous and noisy cell updates. As proposed by Mordvintsev *et al.* (*46*), relaxing the traditional assumption of synchronous update in CA (which assumes a global clock) is closer to what you would expect from a self-organized system, and can be done by applying a random update mask on each cell (parameterized by the update mask rate in Fig. 4B). Despite the update mask enforcing asynchronous and less (or more) frequent cell updates at test time, the discovered parameters still give rise to self-organized moving solitons that perfectly self-maintain (survival scores of one) and that showcase very similar morphology and behavior as the case of synchronous updates (movie S14). Such patterns move slightly slower (or faster), but this is what we can expect as each cell is updated in average only a fraction of the time (or several times per timestep). We also relax the assumption of exact update by adding random noise, of various amounts and magnitudes, to the cell states during the system rollout. Here, we observe that the discovered patterns can resist quite consequent quantities of noise, but past a certain level, as expected, the collective loses its integrity and disintegrates (Fig. 4B).

#### Changes of initialization

While the initialization pattern has been learned with a lot of degrees of freedom (pattern in ${\left[0,1\right]}^{40\times 40}$ ), we can look whether similar patterns (phenotypes) can self-organize from other (maybe simpler) initialization patterns. Biological organisms are able to maintain phenotypic stability in the presence of important perturbations of their embryo structure (*51*), and we can expect a similar property in our system as robustness to changes in the initialization state.

As shown in Fig. 4C, we can see a quasiperfect robustness to noise-altered initial states, and this even for quite high amounts of noise (except for a few configurations that lead to death). These results suggest that the final phenotype forms a strong attractor toward which the different initial mass patterns tend to converge under the learned CA rule. The learned CA rules are hence prone to encode, grow, and maintain a specific target morphology (and its associated functionality), which is consistent with the sensorimotor agent ability to recover from obstacle-induced perturbed morphology. As illustrated in Fig. 5B, we also tested for handmade initial patterns such as bigger disks and same-size asymmetrical disks (for example, with gradient activation). The large disk initialization led to multiple entities forming and separating from each other. The same-size disk, which is much simpler than the trained initial states (but preserves some form of asymmetry) also converged toward the same morphology. However, the robustness to initialization is not perfect as many initializations, such as a disk of smaller size and/or without asymmetry, easily lead to death (movie S18).

#### Changes of scale

Similarly, while the initialization and update parameters have been learned at a certain spatial resolution during training, resulting in self-organized patterns of a certain size (in terms of number of cells), we can artificially change the scale at test time by approximate resizing of parameters (see section S8.5). As shown in Fig. 4D, we tested for different down-scaling (and up-scaling) factors that resulted, for most of them, in fully functional sensorimotor agents with the overall same structure but smaller (or larger) size in terms of number of cells. For sensorimotor agents that are down-scaled and, hence, have much less pixels/cells to do the computation, it is particularly unexpected that they are still able to sense and react to their environment and still show relatively advanced levels of robustness (movie S15). This scale reduction has a limit (a scaling of 0.15 already leads to some death), but we can go quite far down and still obtain functional phenotypes. For the size-increased patterns, which therefore have more space to compute (but also more cells to organize), we observe similar results where sensorimotor agents still self-organize to functional phenotype. Once again, this resonates with findings in biology suggesting that organisms are able to accommodate cell-size differences by adjusting the cell number to maintain a roughly constant body size and structure (*52*).

#### Interactions

We were then interested to test how the discovered sensorimotor agents would react when interacting with other agents in the grid. Given the set of parameters ( $A_{l},\theta_{l}$ ), we can trigger the forming of several macroentities at test time by replicating the initialization square pattern ( $A_{l}\in {\left[0,1\right]}^{40\times 40}$ ) at different locations within a larger grid ( $A^{t=1}\in {\left[0,1\right]}^{512\times 256}$ ) and letting the system unroll. Doing so leads to the development of several entities of the same “species” (governed by the same update rule/physic $\theta_{l}$ ). As illustrated in Fig. 5, we did that for several of the discovered sensorimotor agents and qualitatively observed several interesting emergent interactions.

The first thing that we observed is that several of the discovered sensorimotor agents show strong individuality preservation (movie S11). The fact that the individual agents do not merge nor enter in destructive interactions despite being all made from identical cells is an intriguing example of how the boundary of a “self” (*54*) can emerge and maintain in self-organizing systems. In particular, results suggest that, in the Lenia system, individuality can be obtained as a byproduct of training a sensorimotor agent alone. Our intuition is that, by trying to prevent too much growth during training, it learned to prevent any living cell that would make it “too big,” including living cells from other entities here.

A second type of interaction that can be observed with certain parameters/environments is “attraction.” As illustrated in movie S13, two sensorimotor agents placed in the same grid can show attraction when coming close enough to one another, leading them to stay together and move in the same direction. When they encounter an obstacle, they are able to separate briefly and then reassemble together. Similarly, even when they stay together, we can still qualitatively observe two distinct entities that are interacting with one another while maintaining their overall shape and integrity. This type of behavior has been studied in the game of life under the concept of “consensual domain” (*10*).

A third type of interaction that has been observed in some of the discovered sensorimotor agents is a form of “reproduction,” where collision between two patterns gives rise to the birth of a third entity (movie S12). This kind of interaction seems to happen when one of the two colliding entities is in a certain “mode,” like when it just hit a wall. Our intuition is that when it hits a wall, the self-organizing sensorimotor agent produces a growth response to recover. During this growth response, if there is extra additional mass coming from another entity, then the self-organizing sensorimotor agent might split off from the created mass, while the separated mass, from robust self-organization (see the “Changes of initialization” section), grows into a complete individual. The reproduced individual exhibits the same behavior as its “parents,” i.e., it also navigates the field of obstacles (as shown in the movie S12).

#### External control

A central challenge in synthetic biology, when faced with unconventional forms of agency such as collective of cells, is to find new ways to communicate with the cells to induce desired behaviors at the collective level without having to physically “rewire” the structure of the agent (e.g., via genome editing) but rather by introducing externally controlled cues in the environment (*7*). Here, we are interested to see whether we can induce additional target behaviors in the discovered sensorimotor agents without having to modify the learned parameters $\theta_{l}$ . In particular, we investigate whether the sensorimotor agents can show attraction to some novel elements in their environment (like in nature organisms, being attracted to certain chemicals, lights, or temperatures) and whether we could use those elements to guide the macroentity. To do so, we introduce an additional type of “attractive” low-level elements within the Lenia CA paradigm. More precisely, given the set of learned parameters $\theta_{l}$ , we introduce an additional local rule with parameters $\theta_{a}$ that determine the physical influence of the attractive elements onto the pattern cells. To find parameters $\theta_{a}$ triggering the desired attraction effect at the agent behavioral level, a simple random search with automatic prefiltering and final human assessment was performed (see section S8.5 for details on the procedure). Movie S17 is an example of obtained behavior where we can clearly see that the sensorimotor agent is getting attracted to the newly introduced environmental element (disk of cyan particles), which allows the external user to “control” the sensorimotor agent trajectory by moving the disk in the grid. In spite of this additional behavior, agents are capable to maintain their normal sensorimotor capabilities, showing robustness to collision with obstacles and other agents in the grid. Besides, once the attractive element is removed, the sensorimotor agents return to their normal behavior. However, adding extra rules also weakens the equilibrium that existed in the sensorimotor agent rules, as it creates perturbations that the agent has not been trained to withstand, leading sometimes to death or explosion (or to other behaviors such as reproduction due to extra boost of growth). Once again, parallels can be drawn with findings in biological organisms, for instance, (*55*) shows that controlled ultraviolet light beam can be used to externally guide the trajectory of microswimmers to perform on-demand drug discovery. While we only tested for the attraction type of generalization behaviors, we believe that more sophisticated types of environmental guidance could be induced, although probably necessitating more advanced search methods.

#### Morphological computation

In the current system, there are no direct predefined sensing signals from the obstacles to the pattern in the learnable channel. Instead, this pattern should detect obstacles through the deformations they induce on its own structure. This can be viewed as a form of morphological computation (*20*) where behavior results from a local perturbation, inducing a reorganization of the morphology driven by the interaction of decentralized entities at the micro level (in contrast to a behavior implemented in a central controller such as the central nervous system). To illustrate this mechanism and better understand how the sensorimotor functionality operates, we conduct an experiment without obstacles where we manually remove a portion of a self-organized pattern identified through our search (Fig. 5G and movie S16). We qualitatively observe that the sensorimotor agent reconstitutes its morphology while changing direction in response to this perturbation, i.e., obstacle avoidance is implemented as a morphological computation. The reconstituted pattern still preserves its sensorimotor capabilities as shown in movie S20.

## DISCUSSION

While some basic behavioral capabilities (spatially localized and moving entities) had already been found in Lenia with random search and basic evolutionary algorithms, this work makes a step forward showing how Lenia’s low-level rules can self-organize robust sensorimotor agents with strong adaptivity and generalization to out-of-distribution perturbations. We believe that this contributes to bridging the gap between the enactivist and mechanistic frameworks described in the introduction. While our proposed system is radically enactivist, i.e., where macro-level behavior is an emergent property of micro-level interactions, the resulting behavior resembles what could be observed with mechanistic agents preequipped with sensors, actuators, and decision-making abilities [e.g., reinforcement learning agents (*56*)]. Our main contribution resides in the fact that, in our system, this sensorimotor functionality is realized at the macro (group) level, showing how a group of simple identical entities can make “decision” and “sense” at the macroscale through local interactions only and without any preexisting notion of body/sensor/actuator. From a subjective perspective, the discovered patterns seem to behave as actual mechanistic agents with a body, sensors, and actuators, whereas they are in fact made of tiny parts all behaving under the same rules. We propose that such patterns exhibit a primitive form of sensorimotor agency in the sense that they are able to self-constitute their own morphology, self-maintain their own individuality, and implement a behavioral functionality in interaction with their environment.

To discover such patterns, this work provides a systematic method based on gradient descent, diversity search, and curriculum-driven exploration to easily learn the update rule and initialization state, from scratch in high-dimensional parameter space, leading to the systematic emergence of different robust agents with sensorimotor capabilities. Moreover, we propose to quantitatively characterize sensorimotor agency through a battery of empirical tests applied to the discovered patterns. We believe that the set of tools presented here can be useful in general to discover parameters that lead to complex self-organized behaviors.

However, our proposed empirical tests are specific to the proposed system, e.g., measuring the ability of Lenia patterns to form stable solitons, to move, and to resist to perturbations induced by the presence of obstacles. Future work shall consider how more general measures of agency and sensorimotor capabilities could be applied to the high-dimensional systems studied here (*26*, *27*).

In addition, engineering subparts of the environmental dynamics with functional constraints (through predefined channels and kernels) has been crucial in this work to shape the search process (*57*) toward the emergence of sensorimotor capabilities, as well as used as a tool to analyze more easily these emergent sensorimotor capabilities. An interesting direction for future work is to add even more constraints in the environment such as the need for food/energy to survive, the principle of mass conservation, or even the need to develop some kind of memory to anticipate future perturbations. We believe that richer environmental constraints and opportunities might be a great leap forward in the search for more advanced agent behaviors, in line with recent proposals on the fundamental principles and constraints of living systems (*58*–*60*). For example, behaviors like competition between individuals/species for food, foraging, or even basic forms of learning might emerge. From this competition and constraints, interesting strategies could emerge as a form of autocurricula, as in (*57*, *61*). Promising steps in this direction have been made in (*62*), which introduces Flow Lenia, a mass conservative version of Lenia. In particular, Flow Lenia also allows several species of patterns to coexist in the same grid, leading to competition for matter between the species.

Beyond individual capabilities, we could even wonder under what conditions one could observe the emergence of an open-ended evolutionary process (*63*) directly in the environment, without any outer algorithm, resulting in the emergence of agents with increasingly complex behaviors like building the physical rules of a “universe” and letting agency and evolution emerge from the interactions between parts. To achieve this, we might need to use an optimization process similar to the one presented in this article to evolve all the environmental rules instead of prespecifying some of them by hand. While the engineering of specific environmental rules facilitates the understanding/studying of the results, having more systematic ways to generate them could take us closer to the fundamental scientific quest of designing open-ended artificial systems with forms of functional life and agency “as it could be.” Some preliminary studies are underway (*64*).

Beyond those fundamental scientific questions, future work might also consider broader applications of this work for biology and AI. In biology, inferring low-level rules to control complex system–level behaviors is a key problem in regenerative medicine and synthetic bioengineering (*65*, *66*). In this regard, CA offers an interesting framework to model, understand, and control the emergence of growth, form, and function in self-organizing systems. However, they remain abstract models: Entities in the CA exist on a predefined grid topology, whereas physical entities have continuous position and speed; states in the CA are well-defined, whereas it is not clear where and how information is processed in living organisms; rules in the CA operate at a predetermined scale, whereas real-world processes operate at nested and interconnected scales. In AI, with the recent rise of web-deployed machine-learning models including large language models (*67*, *68*), we are also faced with an increasing blurring of boundaries between the AI and the rest of the “environment” (human end-users and the web itself). It is hence central to understand how to measure emergent agency and cognition in those AI systems, as well as how to interact with them despite the extremely large input and behavioral spaces involved. In this regard, we believe that environments like the one considered in this work can be useful to better inform the debate in much bigger models, as they are rich enough to support emergent agential behaviors while simple enough to study those questions explicitly. Far from trivial, transferring insights from the considered artificial systems into real biological systems or very large AI systems is an exciting area of research with a potential broad range of medical and societal applications (*69*, *70*).

## MATERIALS AND METHODS

### System

An update in Lenia is given by the different rules composing the function $f_{\theta }$ ; each rule is composed of a convolution kernel (which will sense the surrounding of the cell) and a growth function (a function that will convert this sensing, a scalar, into an update of the mass, another scalar). The update of the cells is then given by a weighted sum of the update given by each rule. At each step, the calculation of the update is done identically on every cell of the grid (every cell applies the same convolution filter and growth function). This update is then added to the associated cell, and the result is clipped between 0 and 1. See Fig. 2 for an illustration of the update. The Lenia system used in this work is slightly different from the one in the original paper (*30*, *31*). We changed the parameterization to allow more gradient to flow through the steps (more details in section S6.1). We also chose to use 10 rules, from the learnable channel to itself. We refer to section S6.3 for details on the parameters of the systems and their role. In total, the 10 rules are controlled by 132 parameters.

### Modeling of environmental constraints

The parameter $\theta_{f}$ gives the update rule associated with obstacles. This rule senses the obstacle channel and updates the learnable channel. This means that the convolution will be calculated upon the obstacle channel and the growth obtained through the growth function will be added to the learnable channel. In practice, for $\theta_{f}$ , we use a rule with a convolutional kernel of small size, so that obstacles have effects only locally and a growth function that has a huge negative decrease of mass in the learnable channel to prevent any matter from going where obstacles are present. More information is presented in section S6.2.

### IMGEP

Our proposed method, based on the IMGEP framework (*35*) and fully described in section S7, starts by initializing the history with 40 random parameters and their associated reached position (position of the center of mass at last timestep) computed over 20 rollouts with random obstacle configurations. The method then begins a loop where each step is composed of (i) the sampling of a goal (*x*, *y* position in the grid) and then (ii) a selection from the history of the parameters reaching the closest goal, which will be used to initialize the parameters, (iii) an optimization of those parameters toward the goal under several obstacle configurations, and (iv) a test of those parameters over 20 obstacle configurations to compute the final reached position after optimization, and adding the couple (parameters, reached position) to the history to reuse it in the next steps. Pseudo-code 1 and fig. S12 illustrating the IMGEP algorithm can be found in the Supplementary Materials. Details for each step of methods (i), (ii), (iii), and (iv) can be respectively found in sections S7.4, S7.8, S7.6, and S7.7.

In this work, the loop defined above is composed of 120 outer steps where one out of five outer steps performs 125 steps of gradient descent while the rest performs random mutation on the initialized parameters and 15 steps of gradient descent (details on mutations in section S7.5). At every gradient descent step (Fig. 2), we run a Lenia rollout with the current parameters ( $\theta_{l},A_{l}$ ) and random obstacle placement ( $A_{f}$ ) for 50 timesteps and apply a mean square error loss between the last state of the learnable channel (at the last timestep) and a disk centered at the position of the goal we want to achieve. The gradient is then backpropagated through the Lenia steps to optimize both the parameters of the rule $\theta_{l}$ and the initialization $A_{l}$ (details in section S7.6). As stated before, the obstacles are placed only on one side of a 256-by-256 grid. In total, at every rollout, eight disks of radius 10 are randomly placed as obstacles.

Note that we filter from the history parameters leading to a collapse (mass reaches 0) and explosion of the pattern (pattern expanding too much) both when initializing the history with random parameters and also after an optimization loop (when the optimization fails) so that we do not use them as a starting point for optimization in next steps. More details on the filter we applied can be found in section S7.8.

As presented before, our IMGEP outputs 160 parameters for each seed: 40 from the initialization of history and 120 from the IMGEP steps afterward (one for each step). We discard the intermediate result of optimization and, in each step of the IMGEP, only save the final result of the optimization.

The initialization of the history plays an important role in the subsequent steps of the methods as all the following steps will be built on top of this basis; see Fig. 3A. We thus introduce an initialization selection to find promising initialization of the history. More details on this initialization selection mechanism can be found in section S7.3. Note that those steps are counted in the total number of Lenia rollouts performed by the method for a fair comparison with random search and are the main source of stochasticity in the number of rollouts performed by a run of the method.

### Robustness evaluation

To measure the robustness of the moving stable solitons against obstacles in the “basic obstacle test,” we run 50 rollouts of 2000 timesteps with different obstacle positions. Each rollout environment has 23 obstacles of radius 10 randomly sampled uniformly in the whole grid and one placed in the trajectory of the moving stable solitons (to be sure that it encounters obstacles); more details are shown in section S8.4. At the end of the 2000 timesteps, we compute statistics on the system rollout to detect whether the matter is considered as a stable soliton. We refer to section S8.1 for more information on the statistics used for empirical agency and robustness tests. We then compute the ratio between the number of rollouts (i.e., environments) where the pattern survived (passed the empirical agency test) and the total number of rollouts. Robustness is measured similarly in the generalization tests but with 10 rollouts instead of 50. See section S8.5 for more information on the different generalization tests.

### Handmade search

The parameters from the original Lenia papers (*30*, *31*) are obtained from https://github.com/Chakazul/Lenia. We filter out the ones with multiple channels and the ones with an initialization that does not fit in the 256-by-256 grid; more details are shown in section S9.2.

## Supplementary Materials

### The PDF file includes:

Supplementary Text Figs. S1 to S12 Tables S1 and S2 Legends for movies S1 to S20 References
