# Supplementary material for: Discovering sensorimotor agency in cellular automata using diversity search
Source: Sci Adv. 2025 Oct 31;11(44):eadp0834. doi: 10.1126/sciadv.adp0834 (PMC12577699; doi:10.1126/sciadv.adp0834)
Supplement: Supplementary file 1 — Supplementary Text Figs. S1 to S12 Tables S1 and S2 Legends for movies S1 to S20 References [file sciadv.adp0834_sm.pdf]

Science Advances
AAAS

Supplementary Materials for

Discovering sensorimotor agency in cellular automata using diversity search

Gautier Hamon *et al.*

Corresponding author: Gautier Hamon, gautier.hamon@inria.fr

Sci. Adv. **11**, eadp0834 (2025)
DOI: 10.1126/sciadv.adp0834

The PDF file includes:

Supplementary Text
Figs. S1 to S12
Tables S1 and S2
Legends for movies S1 to S20
References

Other Supplementary Material for this manuscript includes the following:

Movies S1 to S20

- In the first part of this appendix we provide several additional results :
  - In section S2, we provide the resulting curriculum “phylogeny” from a run of IMGEP.
  - In section S3, we provide ablation of the IMGEP method: removing obstacles from the training in S3.1, replacing the gradient with a simple evolutionary algorithm in S3.2, and replacing the biased goal sampling by an uniform goal sampling in S3.3.
  - In section S4, we provide results for each of the 10 seeds to display the variability.
  - In section S5, we provide the full results for the generalization tests.
- We then provide the details of the method, system and tests :
  - In section S6, we describe the Lenia system in details. In particular in subsection S6.1, we describe the change made on the original lenia system from (*30, 31*) to make it more differentiable.
  - In section S7, we describe the IMGEP method in details.
  - In section S8, we provide details about the tests and measures used in the main papers: empirical stable soliton test in S8.1, moving test in S8.2, speed measure in S8.3, basic obstacle test in S8.4, generalization tests in S8.5.
  - In section S9, we provide details about the baselines we use for comparison: random search in S9.1, patterns from the original lenia papers (*30, 31*) in S9.2
- We provied in section S10 the legends of the movies.

S1 Data availability

The resulting parameters as well as their measured performances on the tests tasks are available on Zenodo at https://zenodo.org/records/10211741 in the data folder. More precisely:

- Folder *imgep_exploration* contains parameters generated by the IMGEP method presented in the main text as well as their measured robustness.
- Folder *random_exploration* contains parameters generated by random exploration as well as their measured robustness.
- Folder *handmade_exploration* contains parameters from the original Lenia papers (*30*, *31*) (more details in appendix S9.2) as well as their measured robustness.
- Folder *imgep_no_grad_init_exploration* contains parameters obtained from the IMGEP with ablation on the gradient (described in appendix S3.2) as well as their measured robustness.
- Folder *imgep_no_obstacles_exploration* contains parameters obtained from the IMGEP with ablation of the obstacles (described in appendixS3.1) as well as their measured robustness.
- Folder *imgep_random_sample_init_exploration* contains parameters obtained from the IMGEP with a uniform sampling of goals (described in appendix S3.3) as well as their measured robustness.
- Folder *videos* contains all video presented in this work.
- File *creatures_categories.json* contains the result of the stable soliton and moving test for all the pre-filtered parameters (more details on the pre-filter in appendix S8.1) from the IMGEP, random, handmade exploration and ”IMGEP no obstacles”.

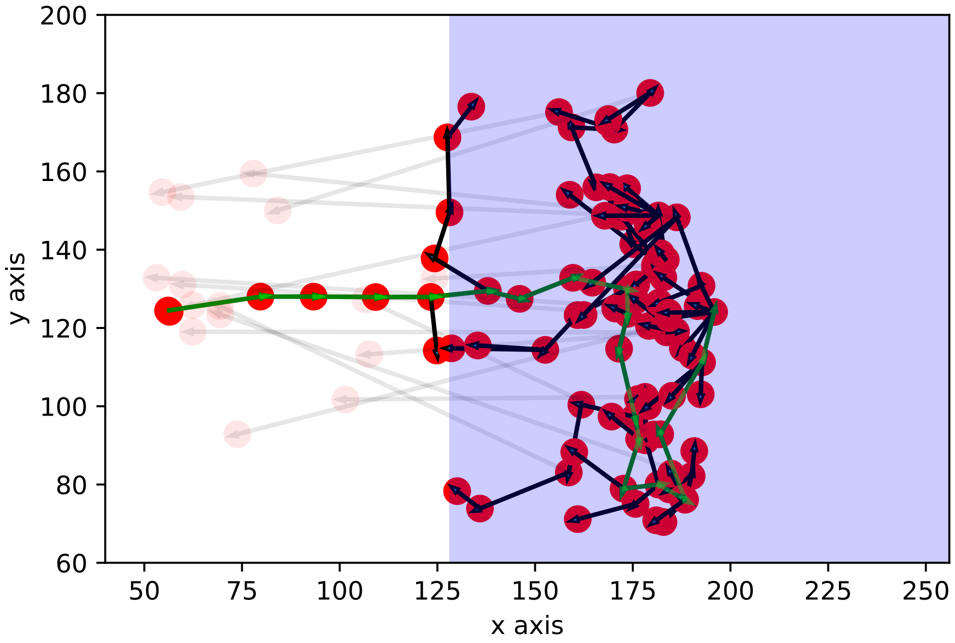

Figure S1: “Phylogeny tree” of one run of IMGEP. The red dot are reached positions (by a step of IMGEP). The blue zone correspond to the zone where obstacles can be placed. Black arrows indicate optimization progress (the point at the end of the arrow was obtained after optimizing the one at the start of the arrow). The path leading to the ”best pattern” (reaching the furthest position on the x axis) is highlighted in green. Interestingly we can see that the best path is not necessarily a straight path. For visibility reasons, we put transparency on the optimization steps that led to reached positions far from the reached position of the parameters that was used to initialize the optimization (often due to failing).

- File *creatures_categories_ablation.json* contains the result of the stable soliton and moving test for all the pre-filtered parameters (more details on the pre-filter in appendix S8.1) from the ablations presented in appendix S3.2 and S3.3.

We also provide the code to reproduce the experiments on Zenodo at `https://zenodo.org/records/10211741`.

S2 Curriculum phylogeny

In Fig.S1, we explore the curriculum path that is generated by the IMGEP. For this aim, we plot the achieved position (reached goal) by each step of the IMGEP. Arrows show, for each step, what was the previous step result used as initialization. In addition, we highlight in green the sequence of reached positions leading to the furthest position attained. We observe that the path to this furthest position is far from being straightforward. This indicates a rather complex optimization landscape toward this position, that would have been difficult to navigate through gradient descent alone. By generating diverse goals and their associated solutions in parameter space, the IMGEP is able to explore potential stepping stones that can later on prove useful to reach difficult positions.

S3 Ablations

We will call the training procedure described in the main text as the *original method*, to which we provide additional detail in S7. In this section, we provide ablation studies aiming to evaluate the effect of removing different components of this original method. To make it as fair as possible and also highlight the difference each ablation introduces, all ablation studies except the "IMGEP no obstacle" were made starting with the same initialization of the history as the ones obtained from the initialization search (S7.3) of the original method. This initialization might however be influenced by the presence of obstacles, this is why "IMGEP no obstacle" will run its own initialization search.

S3.1 IMGEP no obstacles

In this ablation, we use the same training procedure as in the original method but remove the obstacles from the grid. This means that during training, the patterns will only be trained to go further but will never encounter any obstacle.

With this ablation, we obtain moving solitons that are faster without obstacles than the

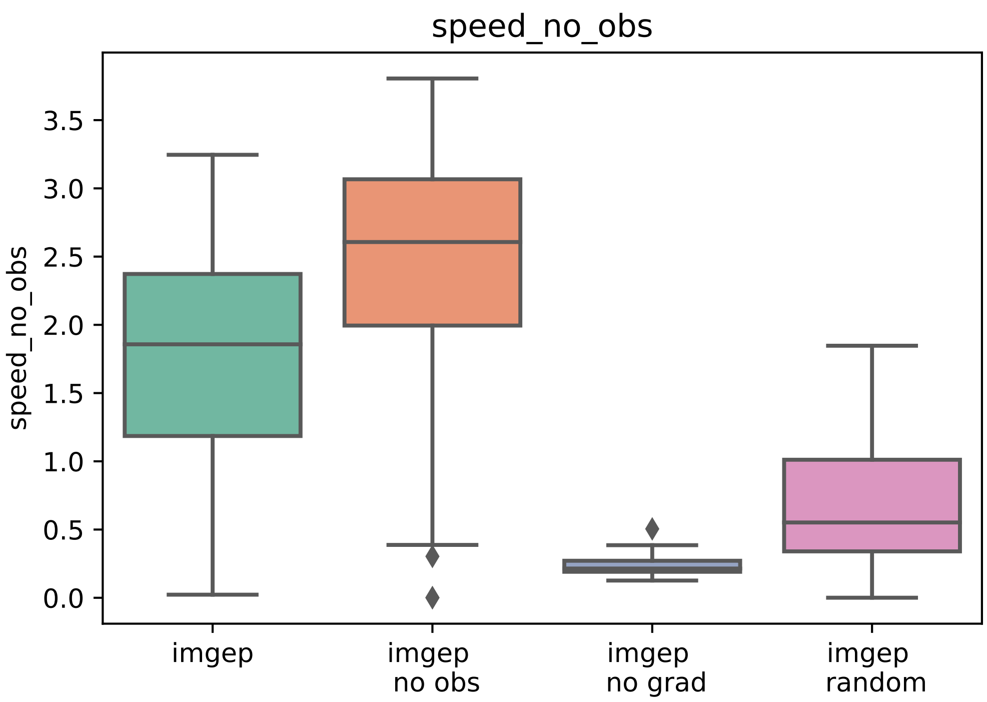

Figure S2: Comparison of ablation on speed

original method (Fig. S2) but have far less robustness to obstacles (Fig. S4) and especially here against moving obstacles (Fig. S5,S6). We also observe that patterns trained in the original condition, at equal speed, are more robust to obstacles than those in this ablation (Fig.S7). This is intuitive as the training without obstacles facilitates reaching further positions (as there is no obstacle in the grid), resulting in higher speed since the episode duration remains constant. However as they are not optimized to resist obstacles, we observe much lower robustness.

S3.2 No gradient

In this experiment, we replace the gradient descent in the original method by a simple evolutionary strategy. For each goal we replace the gradient descent by several parallels trials of random mutation (mutation as described in S7.5) from the candidate parameters with a number of trials equal to the number of gradient descent steps performed during optimization in the original

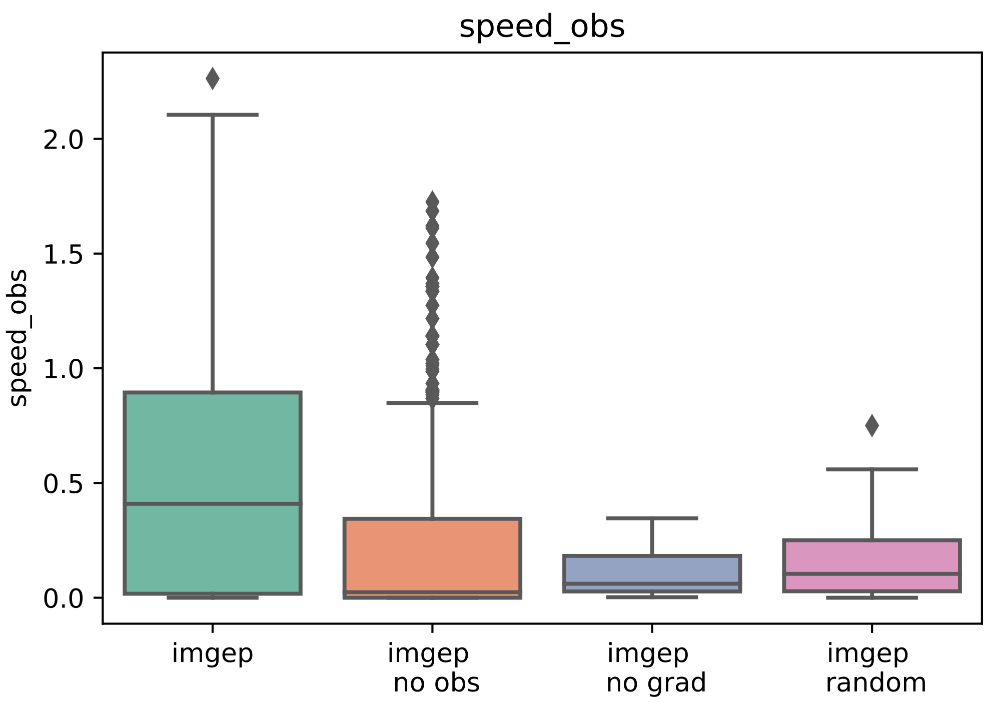

Figure S3: Comparison of ablation on speed with obstacles

method. At the end of those trials we select the parameters having the lowest loss regarding the goal (same loss as the one used for gradient descent in the original method). We observe that the performances of this method is substantially lower (Fig.S8), suggesting that random mutations is not effective in such hard optimization landscapes (and especially with such little number of rollouts) and leads in most cases to explosion or vanish of the matter.

S3.3 Uniform Random sampling of target in IMGEP

In this experiment, we replace the curriculum-driven goal sampling of the original method (detail on curriculum in S7.4) by a uniform sampling in the grid.

Compared to the original method, we observe overall lower performances in term of speed and robustness (Fig. S9 and Fig.S2,S3,S5,S6). This can be explained by the fact that random sampling often sample goals that are impossible to reach at the time. We observe that, with

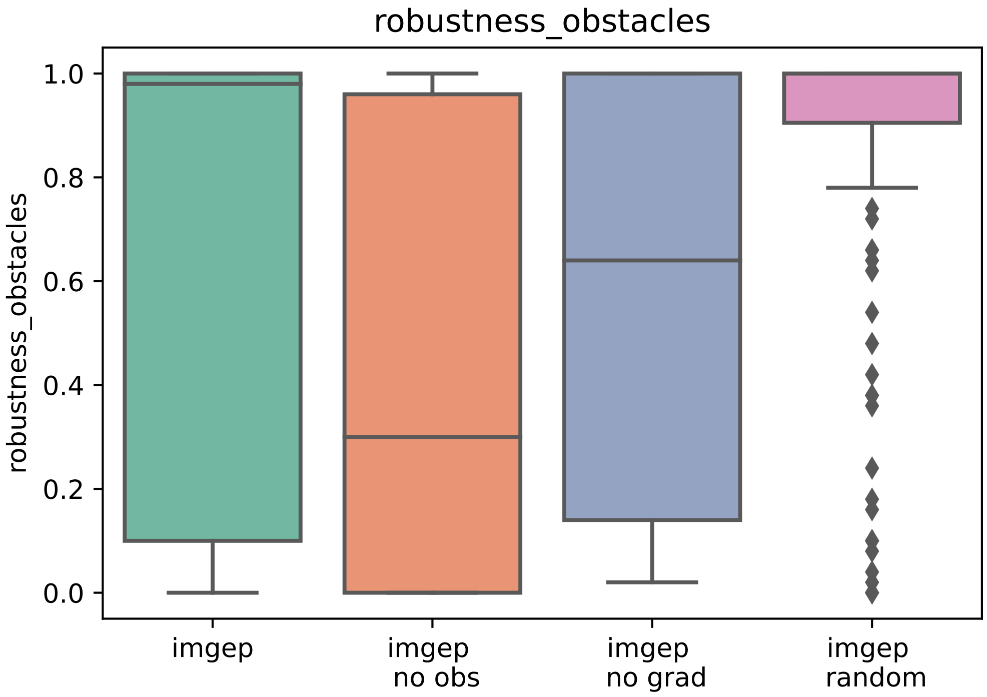

Figure S4: Comparison of ablation on robustness to static obstacles

the same budget as the original method run, it only reaches a very small subset of the entire grid compared to the original method (Fig.S10). Most target goals far from initialization failed while goals that were close enough were sometimes successful.

However, we observe that this ablation still allows to obtain more moving solitons than random search (110 vs. 30)

We introduced a curriculum in our original method mostly to speed up computation. We indeed show with this ablation how it benefits the search process. Note however that, in theory, the current ablation should obtain similar results if given enough compute budget (but will most likely require much more time). In fact, a curriculum can also emerge with random goal sampling, as the patterns will only make progress on goals that either not too far or too close from its current abilities. (see e.g. Forestier et al. 2022 (*35*)).

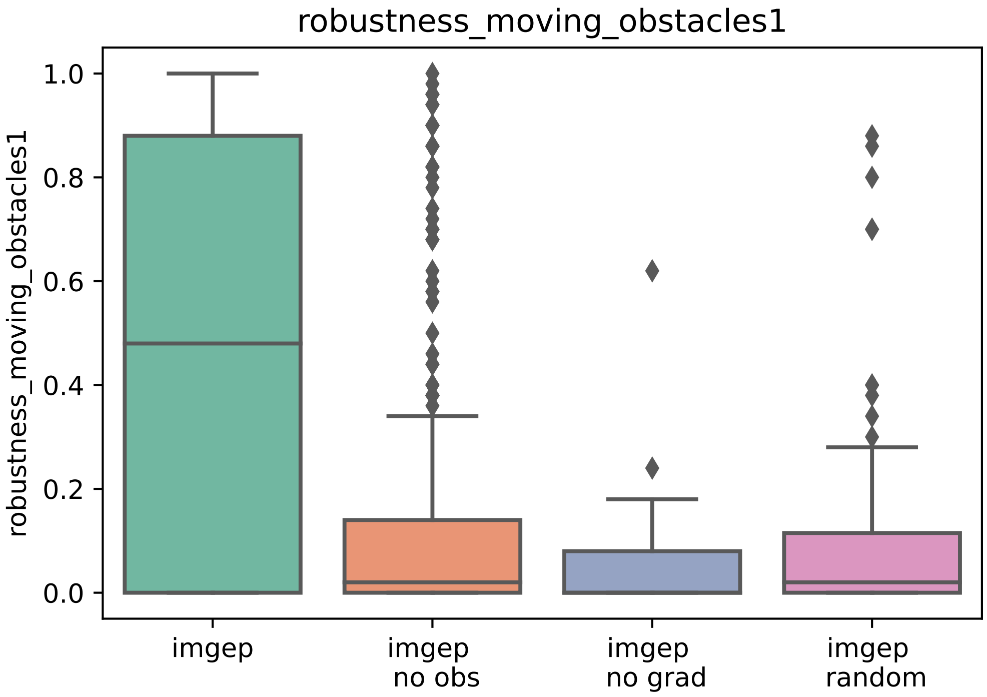

Figure S5: Comparison of ablation on robustness to moving obstacles of speed 1

S4 Seed variability

We report in tab.S1 the variability of the results of the method across the 10 seeds. The variability in result might indicate that some parameter area are easier to navigate or more prone to certain behavior. Overall we still observe that every seed finds a good amount of moving solitons and most of them find at least 1 sensorimotor agent(ie a moving stable soliton with a score ≥ 0.95 to the "basic obstacle test").

S5 Generalization table

We refer to table S2 for the full generalization results.

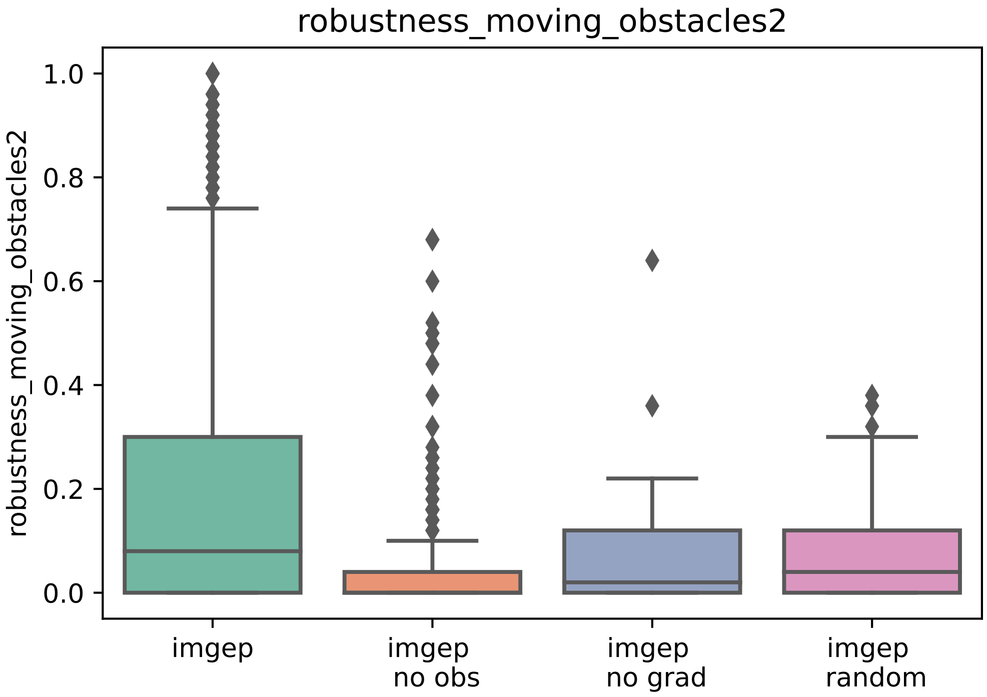

Figure S6: Comparison of ablation on robustness to moving obstacles of speed 2

S6 Lenia system

Cellular automata are, in their classic form, a grid of "cells" $A = \{a_x\}$ that evolve through time $A^{t=1} \longrightarrow ... \longrightarrow A^{t=T}$ via local "physics-like" laws. More precisely, the cells sequentially update their state based on the states of their neighbours: $a_x^{t+1} = f(a_x^t, \mathcal{N}(a_x^t))$, where $x \in \mathcal{X}$ is the position of the cell on the grid, a_x is the state of the cell, and $\mathcal{N}(a_x^t)$ is the neighbourhood of the cell. The dynamic of the CA is thus entirely defined by the initialization $A^{t=1}$ (initial state of the cells in the grid) and the update rule f (function that takes a scalar and outputs a scalar,control how a cell updates based on its neighbours). But predicting their long term behavior is a difficult challenge even for simple ones due to their chaotic dynamics.

Lenia is a class of continuous cellular automata (CA) where each CA instance is defined by a set of parameters θ that conditions the CA rule f_θ; once the parameters θ conditioning the

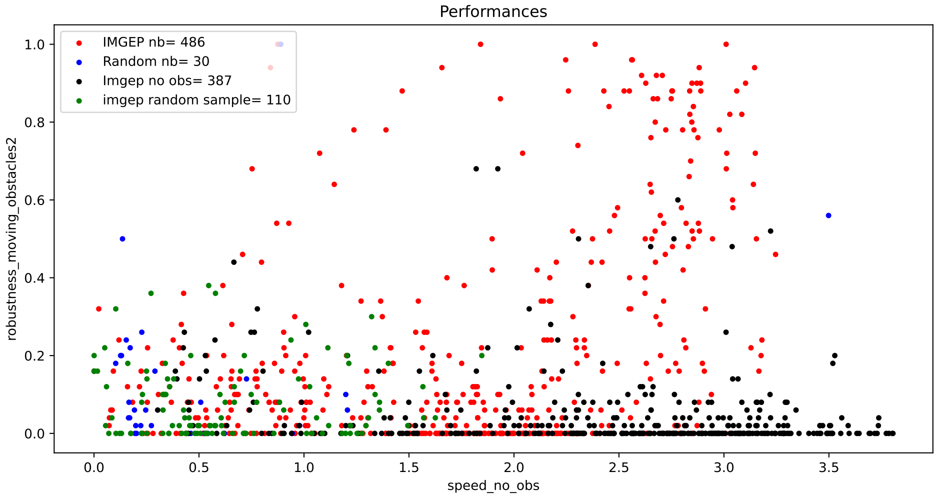

Figure S7: Scatter plot of robustness to moving obstacles of speed 2 (y) and speed without obstacles (x) of IMGEP (red), IMGEP without obstacles in the search (black), Random search (blue) and IMGEP with random sampling of goals (green). Even for moving solitons with comparable speed without obstacles, IMGEP with no obstacles has far less robustness to obstacles of speed 2 than IMGEP trained with obstacles.

update rule has been chosen, the system is a classical CA where the initial grid pattern $A^{t=1}$ will be updated.

In Lenia, the system is composed of several communicating grids $A = \{A_c\}$ which we call channels. In each of these grids, every cell/pixel can take any value between 0 and 1. Cells at 0 are considered dead while others are alive. The channels are updated in parallel according to their own physics rule. Intuitively, we can see channels as the domain of existence of a certain type of cell. Each type of cell has its own physics : it has its own way to interact with other cells of its type (intra-channel influence) and also its own way to interact with cells of other types (cross-channel influence).

The update of a cell $a_{x,c}$ at position x in channel c can be decomposed in three steps. First the cell senses its neighbourhood in some other channels (its neighbourhood in its channel, with cells of the same type but also in other channels with other types of cells) through convolution kernels which are filters K_k of different shapes and sizes. Second, the cell converts this sensing

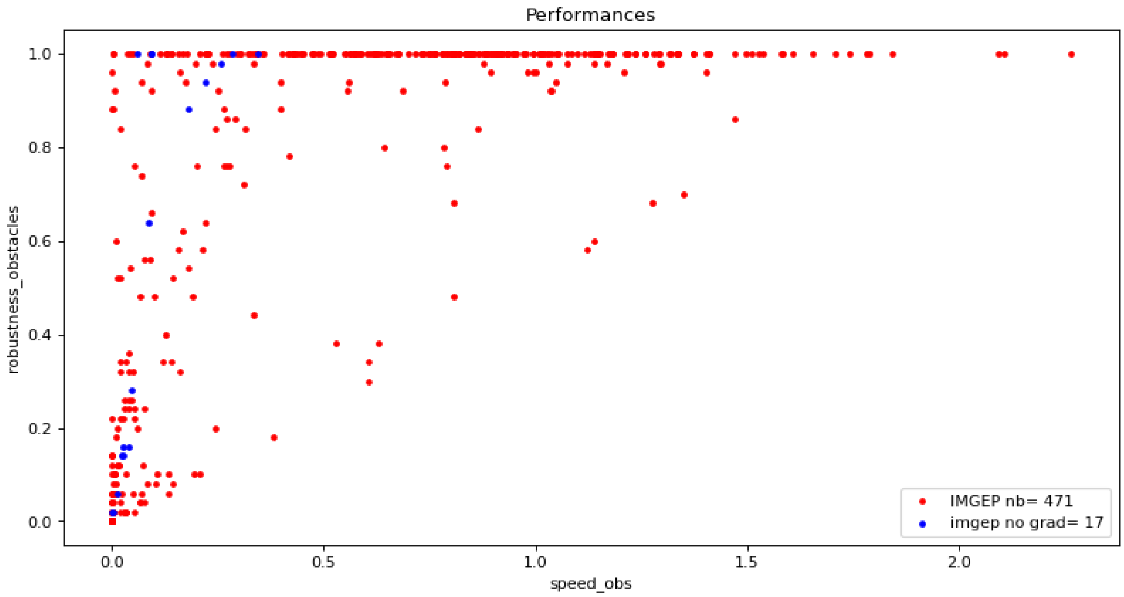

Figure S8: Comparison of the original IMGEP method and an IMGEP where gradient descent optimization of the parameters is replaced by random mutations as described in S3.2. We can see that random mutation hardly succeed in optimizing the parameters leading to very poor performance compared to the IMGEP with gradient descent.

into an update (whether positive or negative growth or neutral) through growth functions G_k associated with the kernels. Finally, the cell modifies its state by summing the scalars obtained after the growth functions and adding it to its current state. After the update of every rule has been applied, the state is clipped between 0 and 1. Each (kernel,growth function) couple is associated to the source channel c_s it senses, and to the target channel c_t it updates. A couple (kernel, growth function) characterizes a rule on how a type of cell c_t reacts to its neighbourhood of cells of type c_s. Note that c_s and c_t could be the same, which correspond to interaction of cells of the same type (intra-channel influence). Note also that we can have several rules, i.e. several (kernel,growth function) couples, characterizing the interaction between c_s and c_t.

A local update in the grid is summarized with the following formula (where G^k, K^k, c_s^k, c_t^k are respectively the growth function, convolution filter, source channel, target channel associated with the k'th rule):

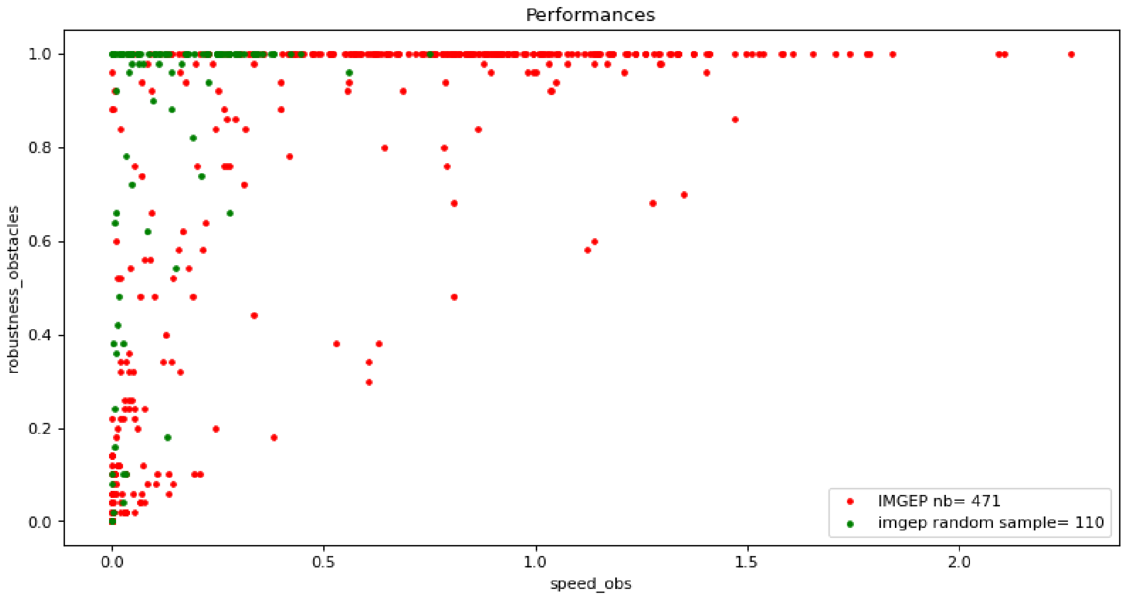

Figure S9: Comparison of the original IMGEP method and an IMGEP where our our biased goal sampling is replaced by a random sampling of goal in the grid as described in S3.3. We can see that our biased sampling is much more efficient at finding robust fast moving sensorimotor agents.

$$a_x^{t+1} = f(a_x^t, \mathcal{N}(a_x^t)) = \begin{bmatrix} a_{x,c_0}^t + \frac{1}{T}\sum_{k \text{ st } c_t^k=0} G^k(K^k(a_{x,c_s^k}^t, \mathcal{N}_{c_s^k}(a_x^t))) \\ \cdot \\ \cdot \\ a_{x,c_C}^t + \frac{1}{T}\sum_{k \text{ st } c_t^k=C} G^k(K^k(a_{x,c_s^k}^t, \mathcal{N}_{c_s^k}(a_x^t))) \end{bmatrix}$$

For each rule, the shape of the (kernel, growth function) is parameterized. We are thus able to "tune" the physics of the cells and of their interactions by changing the kernels shape (how the cells perceive their neighborhood) as well as the growth function shape (how the cells react to this perception).

S6.1 Differentiating through Lenia steps

Due to the locality and recurrence of the update rule, there is a close relationship between cellular automata and recurrent convolutional networks (72). In fact, we can see a rollout in Lenia as applying a recurrent neural network to an initial state. If (some of) the network parameters

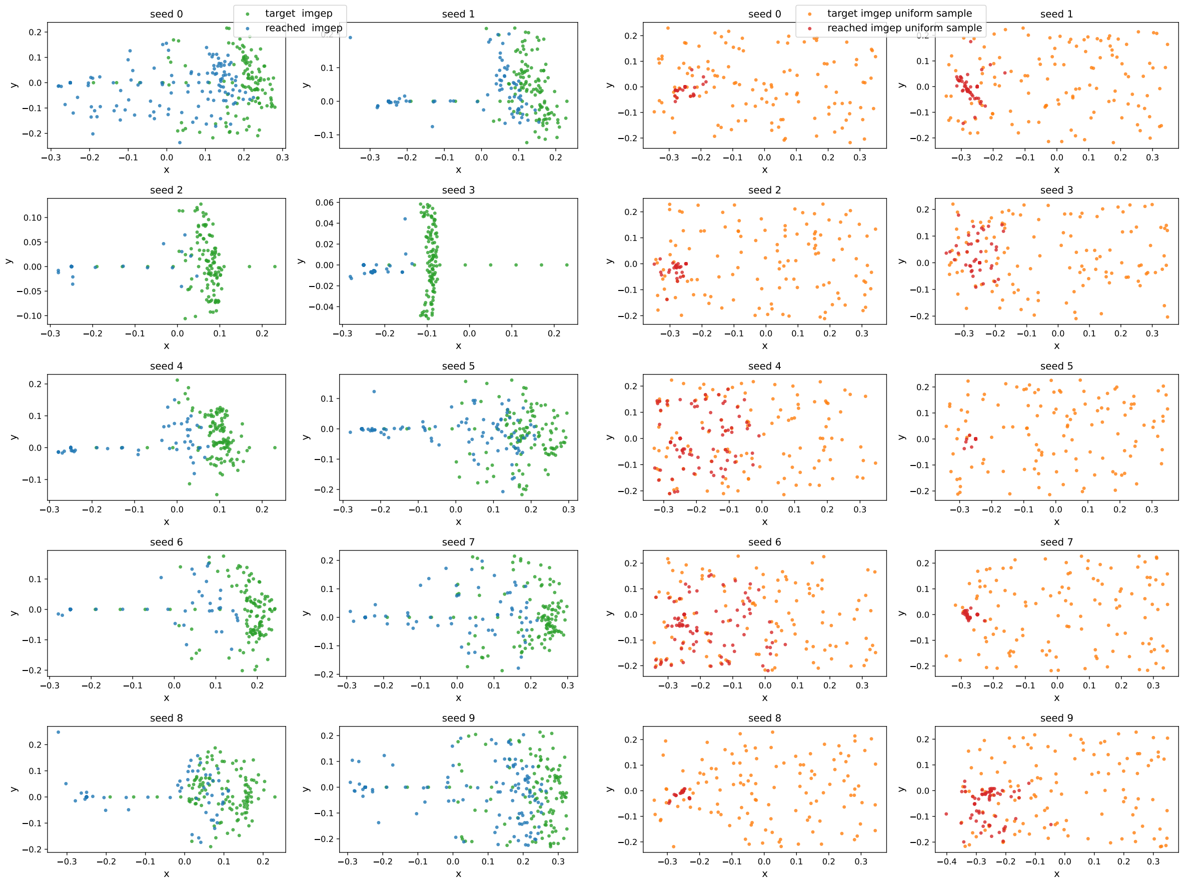

Figure S10: Target goals and reached positions for every seed of (left) original method (right) IMGEP with uniform sampling of goal. The uniform sampling IMGEP sample a lot of far points that not reached at all

are differentiable, backpropagation can be done by "unfolding" the Lenia rollout and applying a loss at certain time step(s) like in (*46*).

However, in the classic version of Lenia, the shape of the kernels are not totally differentiable and not very flexible. To allow easier optimization of the Lenia system, we introduce some changes to the kernel parameterization.

In fact in the original Lenia (*30*), the number of bumps in the kernel (see Fig.S11 left) is fixed and cannot be optimized through gradient descent.

We therefore introduced a class of CA with differentiable parameters. To do so, the main

Table S1: Seed variability

Seed Number	Number of solitons	Number of moving (solitons)	Number of sensorimotor (agents)	max speed (solitons)	max speed obs (soltions)
Seed 0	107	93	91	2.8	1.4
Seed 1	64	54	26	2.7	1.5
Seed 2	33	32	1	2.0	1.1
Seed 3	18	7	0	0.5	0.3
Seed 4	35	26	6	1.9	0.4
Seed 5	66	52	38	2.9	1.8
Seed 6	54	54	2	2.5	0.3
Seed 7	30	30	1	3.0	0.9
Seed 8	44	44	4	2.3	0.3
Seed 9	104	94	92	3.2	2.3

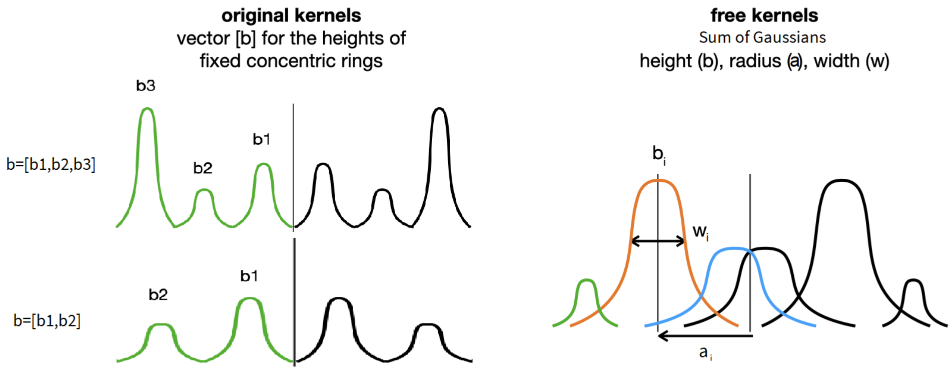

Figure S11: Visualization of (left) the convolution kernels used in the original lenia papers (*30, 31*), (right) the kernel we propose in this paper for more differentiation capabilities. The kernel we propose consists of a sum of free shifted gaussian bumps while the one in the original lenia papers consist of fixed concentrated rings.

Table S2: Generalization results

Tests	IMGEP		Random	Handmade
	speed> 1	10 best	10 best	10 best
speed	1.33 ± 0.28	1.94 ± 0.15	0.53 ± 0.25	0.34 ± 0.10
obstacle				
number				
24	0.98 ± 0.07	0.99 ± 0.03	0.99 ± 0.03	0.99 ± 0.03
30	0.98 ± 0.07	1.00 ± 0.00	0.99 ± 0.03	0.99 ± 0.03
36	0.99 ± 0.06	1.00 ± 0.00	0.99 ± 0.03	0.97 ± 0.09
42	0.99 ± 0.03	1.00 ± 0.00	0.99 ± 0.03	0.97 ± 0.09
48	0.99 ± 0.04	1.00 ± 0.00	1.00 ± 0.00	0.98 ± 0.06
radius				
4	0.92 ± 0.18	0.90 ± 0.13	0.92 ± 0.12	0.95 ± 0.09
7	0.98 ± 0.08	1.00 ± 0.00	1.00 ± 0.00	0.97 ± 0.09
10	0.98 ± 0.07	0.99 ± 0.03	0.99 ± 0.03	0.99 ± 0.03
13	0.98 ± 0.08	0.99 ± 0.03	1.00 ± 0.00	0.99 ± 0.03
16	0.98 ± 0.08	1.00 ± 0.00	1.00 ± 0.00	1.00 ± 0.00
speed				
1/3	0.99 ± 0.04	1.00 ± 0.00	0.77 ± 0.27	0.74 ± 0.28
1/2	0.97 ± 0.07	1.00 ± 0.00	0.61 ± 0.38	0.51 ± 0.38
1	0.81 ± 0.23	0.97 ± 0.05	0.42 ± 0.41	0.02 ± 0.04
2	0.34 ± 0.32	0.71 ± 0.25	0.13 ± 0.29	0.00 ± 0.00
3	0.12 ± 0.15	0.32 ± 0.17	0.07 ± 0.12	0.00 ± 0.00
update				
mask rate				
0.2	0.99 ± 0.08	1.00 ± 0.00	1.00 ± 0.00	1.00 ± 0.00
0.6	0.99 ± 0.08	1.00 ± 0.00	0.89 ± 0.30	1.00 ± 0.00
1.0	1.00 ± 0.00	1.00 ± 0.00	1.00 ± 0.00	1.00 ± 0.00
1.4	0.99 ± 0.09	1.00 ± 0.00	1.00 ± 0.00	1.00 ± 0.00
1.8	0.99 ± 0.10	1.00 ± 0.00	1.00 ± 0.00	1.00 ± 0.00
noise rate				
0.2	0.91 ± 0.28	0.90 ± 0.30	0.77 ± 0.37	0.99 ± 0.03
0.4	0.75 ± 0.42	0.91 ± 0.27	0.74 ± 0.38	0.92 ± 0.18
0.6	0.67 ± 0.45	0.90 ± 0.27	0.58 ± 0.46	0.77 ± 0.38
0.8	0.60 ± 0.47	0.63 ± 0.44	0.50 ± 0.44	0.71 ± 0.44
1.0	0.51 ± 0.47	0.32 ± 0.41	0.44 ± 0.45	0.70 ± 0.46
noise std				
0.2	0.99 ± 0.11	1.00 ± 0.00	0.96 ± 0.12	1.00 ± 0.00
0.6	0.79 ± 0.39	0.90 ± 0.30	0.76 ± 0.39	0.98 ± 0.06
1.0	0.51 ± 0.47	0.32 ± 0.41	0.44 ± 0.45	0.70 ± 0.46
1.4	0.08 ± 0.21	0.03 ± 0.09	0.18 ± 0.32	0.56 ± 0.45
1.8	0.06 ± 0.14	0.06 ± 0.10	0.17 ± 0.30	0.45 ± 0.47
init				
noise rate				
0.2	1.00 ± 0.01	1.00 ± 0.00	0.89 ± 0.16	1.00 ± 0.00
0.4	0.99 ± 0.09	1.00 ± 0.00	0.91 ± 0.24	0.99 ± 0.03
0.6	0.98 ± 0.13	1.00 ± 0.00	0.88 ± 0.30	0.95 ± 0.15
0.8	0.97 ± 0.14	1.00 ± 0.00	0.88 ± 0.30	0.89 ± 0.24
1.0	0.95 ± 0.21	1.00 ± 0.00	0.88 ± 0.30	0.76 ± 0.29
noise std				
0.5	0.97 ± 0.16	1.00 ± 0.00	0.87 ± 0.30	0.97 ± 0.09
1.5	0.94 ± 0.20	0.98 ± 0.06	0.85 ± 0.30	0.52 ± 0.42
2.5	0.89 ± 0.27	0.92 ± 0.17	0.80 ± 0.36	0.37 ± 0.44
3.5	0.86 ± 0.32	0.91 ± 0.27	0.81 ± 0.34	0.35 ± 0.45
4.5	0.85 ± 0.32	0.94 ± 0.18	0.79 ± 0.38	0.32 ± 0.43
scaling				
0.15	0.91 ± 0.28	0.90 ± 0.30	0.30 ± 0.46	0.00 ± 0.00
0.65	0.99 ± 0.10	1.00 ± 0.00	0.50 ± 0.50	1.00 ± 0.00
1.15	1.00 ± 0.00	1.00 ± 0.00	0.70 ± 0.46	1.00 ± 0.00
1.65	1.00 ± 0.00	1.00 ± 0.00	0.70 ± 0.46	1.00 ± 0.00
2.15	1.00 ± 0.00	1.00 ± 0.00	0.60 ± 0.49	1.00 ± 0.00

shift is to use kernels in the form of a sum of k overlapping gaussian bumps:

$$x \to \sum_{i}^{k} b_i exp(-\frac{(\frac{x}{rR} - a_i)^2}{2w_i^2})$$

The parameters controlling the shape are then $3k$-dimensional vectors: b for height of the bump, w for the size of the bump and a for the center of the bump.

These symmetric "free kernels", while very inspired from Lenia's original "vanilla bumps", allow differentiation and more flexibility and expressivity but at the cost of more parameters. For example, it is possible to reduce the number of bumps by assigning some null height values, allowing the number of bumps to be optimized through gradient descent.

In Lenia, a growth function $G : [0, 1] \to [-1, 1]$ is any unimodal non-monotonic function that satisfies $G(\mu) = 1$. In this work, we use the continuous exponential growth function $G(x) = 2\exp\left(-\frac{(x-\mu)^2}{2\sigma^2}\right) - 1$ which is differentiable with respect to μ and σ.

To summarize, the parameters of the update rule are thus those controlling the kernel shape (R, r, a, w, b), those controlling the growth function (μ, σ, h) and a time controlling parameter (T). For a total of n rules (all channels included) with k bumps kernels, the number of parameters is $(3k + 4)n + 2$. In our experiments, R and T are chosen randomly and fixed while all the other parameters are optimized, and we use a total of $n = 10$ rules with $k = 3$ bumps kernels . So in total we have 132 parameters for the rules from which 130 are optimized.

In addition to the rule, parameters we also optimize the initialization square $I_{square} \in [0, 1]^{(40,40)}$.

S6.2 Obstacles

The multi-channel aspect of Lenia allows the implementation of different types of cells/particles. To implement obstacles in Lenia we added a separate "obstacle" channel with a kernel going from this channel to the learnable "creature" channel (see Fig.2.). This kernel triggers a severe negative growth in the pixels of the learnable channel where there are obstacles but has no impact on other pixels where there are no obstacles (very localized kernel). This way

we prevent any growth in the pixels of the learnable channel where there are obstacles. The formula of the growth function is : $G(x) = -clip((x - 1e - 8), 0, 1) * 10$. Hyperparameters of this handmade rule can be found in S6.3.

The learnable channel cells can only sense the obstacles through the changes/deformations it implies on it or its neighbours. In fact, as the only kernel that goes from the obstacle channel to the learnable channel is the one we hand-designed, if a macro pattern emerges it has to "touch" the obstacle to sense it. To be precise the pattern can only sense an obstacle because its interaction with the obstacle will perturb its own configuration and dynamics (i.e. its shape and the interaction between the cells constituting it). This is similar to experiments with swarming bacteria (*73*), where the swarm agent must learn to collectively avoid antibiotic zones (externally-added obstacles) where the bacteria can't live.

In our implementation, obstacles stay still, meaning that there is no rule that goes toward (and hence no update of) the obstacle channel . As such, an update step in the final system is summarized at the bottom of Fig.2..

To test the sensorimotor agents under moving obstacles, we simply shift the channel of obstacles of a certain amount of pixel at every timestep. This shift of the grid, for an integer value of speed, can be written as a rule of the system from the obstacle channel to the obstacle channel. The rule would be the same on all the grid and is localized as it is a function of the fixed neighbourhood. Moving obstacles with a speed with a rational value (for example 0.5 pixels/timesteps) is done in our case by doing the shift every few timesteps.

S6.3 Lenia rules parameters

Here is the list of the parameters associated to the rules of a Lenia system with C channels, nb_k rules with kernels with k bumps. We also provide the range used in this work for the learnable channel. In this work we used C=2 channels (one learnable channel and the fixed channel), $nb_k = 10$ learnable rules and 1 fixed rule (for the obstacles).

- Common to all rules

- $T \in [1, 10]$

- Learnable rules
  - Kernel (convolution filter) parameters:
    - R $\in [15, 40]$ Radius of the kernels (common to all kernels)
    - r $\in [0, 1]^{nb_k}$ relative radius of each kernel.
    - b $\in [0, 1]^{nb_k, k}$ height of the k bumps.
    - w $\in [0.01, 0.5]^{nb_k, k}$ width of the k bumps.
    - a $\in [0, 1]^{nb_k, k}$ position of the bumps on the radius.
  - Growth function $G(x) = 2\exp\left(-\frac{(x-\mu)^2}{2\sigma^2}\right) - 1$ parameters
    - $\mu \in [0.05, 0.5]^{nb_k}$ mean of the gaussian growth function.
    - $\sigma \in [0.001, 0.18]^{nb_k}$ variance of the gaussian growth function.
    - h $\in [0, 1]^{nb_k}$
  - $c_0 = [0] \times nb_k$ source channel (0 is learnable channel)
  - $c_1 = [0] \times nb_k$ destination channel

- Fixed rule
  - Kernel parameters:
    - R = 4 small radius for very localized action
    - r = [1,1,1]
    - b = [1,0,0]
    - w = [0.5,1,1]
    - a = [0,0,0]
  - Growth function $G(x) = -clip((x - 1e - 8), 0, 1) * 10$
  - $c_0 = 1$ source channel (1 is fixed channel)
  - $c_1 = 0$ destination channel

S6.4 Lenia rule parameter mutations

- Common to all rules
  - T : $\mathcal{N}(0, 0.1) \times \mathcal{B}(0.01)$ (mutation then integer)
- Learnable rules
  - Kernel (convolution filter) parameters:
    * R $\mathcal{N}(0, 0.1) \times \mathcal{B}(0.01)$ (mutation then integer)
    * r :$\mathcal{N}(0_{nb_k}, 0.2 \times \mathcal{I}_{nb_k})$
    * b : $\mathcal{N}(0_{3nb_k}, 0.2 \times \mathcal{I}_{3nb_k})$
    * w :$\mathcal{N}(0_{3nb_k}, 0.2 \times \mathcal{I}_{3nb_k})$
    * a : $\mathcal{N}(0_{3nb_k}, 0.2 \times \mathcal{I}_{3nb_k})$
  - Growth function $G(x) = 2\exp\left(-\frac{(x-\mu)^2}{2\sigma^2}\right) - 1$ parameters
    * μ : $\mathcal{N}(0_{nb_k}, 0.2 \times \mathcal{I}_{nb_k}) \times \mathcal{B}(0.1)$
    * σ: $\mathcal{N}(0_{nb_k}, 0.01 \times \mathcal{I}_{nb_k}) \times \mathcal{B}(0.1)$
    * h $\mathcal{N}(0_{nb_k}, 0.2 \times \mathcal{I}_{nb_k}) \times \mathcal{B}(0.1)$

S7 IMGEP details

Algorithm 1 IMGEP pseudo code

Initialization: history $\mathcal{H}$ and models $\mathcal{T}, \Pi, Optim, R$.
for i=1..N **do**
Generate a target goal $\tau_i \sim \mathcal{T}(\mathcal{H})$ ▷ use of *curriculum learning* and *diversity search*
Train parameters on target goal $\theta_i^* = Optim(\theta_i|\tau_i)$, where $\theta_i \sim \Pi(\mathcal{H}|\tau_i)$ ▷ use of *gradient descent* and *stochasticity handling*
Evaluate parameters $x_i \sim R(\theta_i^*)$ ▷ *behavioral characterization*
Store in history $H \leftarrow H \cup (\theta_i^*, x_i)$ ▷ *reuse knowledge* for task sampling and training
return $\mathcal{H}$

In this section, we first recall the basics of the IMGEP procedure and then go into the details of each element of the method.

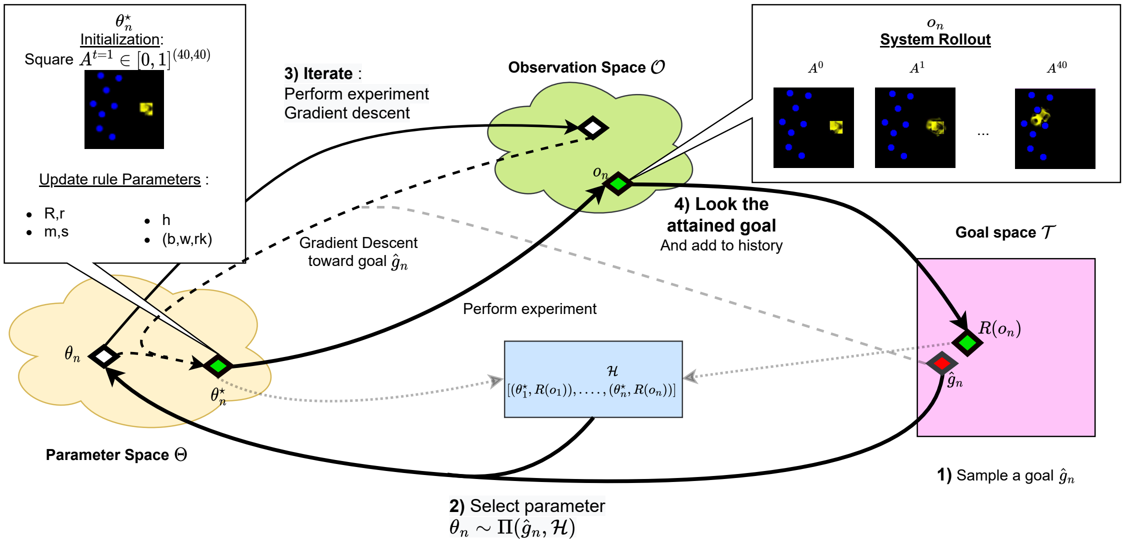

Figure S12: IMGEP loop

Our method described in the pseudo code 1 starts by initializing a pool of (parameters, reached position) couples by random search, this constitutes the initial state of the history $\mathcal{H}$ (details in S7.1). Then, at each iteration, the method iterates through the following steps (illustrated in Fig. S12). **1) Sample a new goal** using a goal sampling strategy which takes into account the previously reached positions (details in S7.4). An example of the sampling distribution can be found in green in Fig 3.a. **2) Infer starting parameters for that goal** by selecting parameters $\{(\theta_l, A_l^{t=1})_i\}_{i=1...t-1}$ associated to a previously reached position in history $\mathcal{H}$ that is close to the sampled goal (details in S7.8). **3) Optimize parameters toward the sampled goal** by iteratively performing rollouts of the Lenia system under different environmental conditions A_f and applying stochastic gradient descent on the MSE loss between the disk at goal position and the mass of the learnable channel at the last timestep (details in S7.6). **4) Update history** $\mathcal{H}$ with the newly obtained parameter point and test it in various environmental conditions A_f to estimate its reached position (details in S7.7) (such that it can be later reused as a starting point for achieving other sampled goals).

As described in the main text, the behavioral space is the position (x,y) of the center of

mass at the last timestep of the rollout. The loss we use is the Mean square error loss between the learnable channel at the last timesteps of the rollout and the same grid with a superposition of 2 disk centered at the goal position in the first channel. The target disk has this formula: $0.9x(0.15x(R_g < 10) + 0.85x(R_g < 5))$ where R_g is the euclidian distance to the goal position.

To introduce more diversity in the search (and potentially getting out of difficult optimization landscape), some steps of IMGEP add mutation to the promising parameters before applying the optimization through gradient descent. More details can be found in S7.5.

Note that we also introduce an automatic way for the method to restart again from scratch in case of not good enough first steps (not present in pseudocode 1). We refer to subsection.S7.3 for a detailed description of this restarting mechanism.

Note that the goal positions as well as the measured reached positions (details in S7.7) are normalized and centered between -0.5 and 0.5 (so that obstacle positions are at $x > 0$,Fig.3 in the main text) according to the map size SX.

The following sections provide additional details about different parts of the method.

S7.1 Intialization of history

The IMGEP method first applies an initialization of history $\mathcal{H}$ through random search to bootstrap the whole IMGEP procedure.

In this work, the initialization of history consist of 40 trials of random parameters. The range used for this random search are the one presented in S6.3 except that we divide the strength of the kernels parameters h by 3. This change is done in order to have weaker/slower updates increasing the chance to have a pattern not exploding or vanishing in 50 timesteps, in order to facilitate further optimization.

This dividing of h by 3 is only to make things go faster (requiring less trials for the initialization of history) with some human heuristic on the system but should not be mandatory as random search without this should also get interesting parameters for initialization with more trials.

S7.2 Warming up goal sampling

To accelerate the curriculum, we start the first 8 steps of the IMGEP with a deterministic goal sampling which tries to go as far as possible on the x axis. The goal position starts at position (-0.19,0) and is shifted of +0.06 along the x axis for every of those deterministic steps. The rest of the goal sampling is stochastic as described in S7.4.

S7.3 Initialization selection

History initialization and the first IMGEP steps have a huge impact on the performance of the method, as it will provide the basis for all subsequent optimization. History initialization and the warm up of goal sampling have a huge impact on the performance of the method, as it will provide the basis for all subsequent optimization.

To mitigate this problem, we also apply initialization selection with the objective of facilitating further optimization. We run the first steps of the method (random initialization and few steps of optimization), and observe the loss for the 3 first deterministic targets (described in section S7.2). If this loss is above a certain threshold for one of the 3 step, we start over again getting rid of the initialization history and initializing it again with random search. We perform this until we find a "good" initialization that is below the threshold for the 3 steps.

S7.4 Goal sampling

The goal sampling we chose in this work intends to sample goals ((x,y) positions) that should be most of the time further in the grid (for harder goals), not too far from previously reached positions (for feasibility of the goal) and also not too close from previously achieved goals (to make progress) . From those heuristic we introduce our engineered goal sampling strategy in pseudo code 2. The objective of this engineered sampling is to accelerate the search but much simpler ones could work if given enough computational budget (see ablation with totally random sampling S3.3).

Algorithm 2 Goal sampling strategy

Input: history $\mathcal{H}$
nb_close=0,nb_veryclose=0
while nb_close¡1 or nb_veryclose ¿2: **do**
  if rand $\sim \mathcal{U}(0,1)$ ¡0.2 : **then**
    goal = bestgoal($\mathcal{H}$)+($\mathcal{U}(0,1) \times 0.04 + 0.02$, $(\mathcal{U}(0,1) \times 0.45 - 0.22)/4$,) ▷ Try little further than previous best
  else
    if rand $\sim \mathcal{U}(0,1)$ ¡0.7 : **then** ▷ Try random far points
      goal=($-\mathcal{U}(0,1) \times 0.2 + 0.35$, $-\mathcal{U}(0,1) \times 0.45 - 0.22$)
    else
      goal=($-\mathcal{U}(0,1) \times 0.35 + 0.35$, $-\mathcal{U}(0,1) \times 0.45 - 0.22$)
  nb_close,nb_veryclose=calc_distances(goal,$\mathcal{H}$)
return goal

S7.5 Mutation

We apply mutations on candidates parameters in order to increase diversity. Some mutations can facilitate optimization while others can lead to undesirable configurations impairing it. For this reason, we apply less gradient steps on those mutated parameters. See section S7.9 for the hyperparameters in this work.

In addition, we generate mutations of a parameter configuration until it results in a pattern not collapsing after 50 timesteps. For this (approximate) collapsing measure, we use a simple soft filter checking if the total mass in the learnable channel at the last timestep is > 10 (to test for death of matter) and if the mean square error between the learnable channel at the last timestep and the disk defined in S7 centered on the center of mass of the learnable channel is < 25 (as a proxy for explosion of the mass, more details in S7.7). This loop of mutations is counted in the total number of rollout performed by the IMGEP.

We refer to section S6.4 for the mutation (distribution, mean, variance) applied to each parameters in the method.

S7.6 Gradient descent

Differentiating through Lenia can be difficult because the gradient must backpropagate through several steps (which moreover have their result clipped between 0 and 1) without vanishing. We should thus limit ourselves to a few iterations when training: in our experiments the loss is applied after 50 steps in Lenia.

Obtaining gradients that are informative for optimization requires an overlapping between the mass in the learnable channel and the disk centered at the goal position. The curriculum we introduce in the goal sampling procedure (S7.4) facilitates this overlap by generating goals that neither too far nor too close from the initial pattern at t=0 and from previously reached goal.

We refer the reader to appendix section A.S3.2 for an ablation of the gradient descent showing the importance of it in the method.

S7.7 Parameter evaluation

We perform an evaluation of the parameters after each IMGEP step (sampling of goal and optimization of parameters). This evaluation consists of running 20 rollouts of 50 timesteps (the same rollout length as in the optimization rollout) with different random obstacle configurations and measures the average reached position over those rollouts.

For each rollout, we also compute the mean square error between the learnable channel at the last timestep and the disk shape centered on the center of mass of the learnable channel at last timestep. We then take the average value over the rollouts. This is used as a proxy "collapsing measure" (explosion or death of the pattern) to apply a soft filter when selecting promising initialization parameter for a new goal as explained in section S7.8.

The parameters (A_l, θ_l), the measured reached position (r_x, r_y) and collapsing proxy measure c are then stored in the history $\mathcal{H}$.

S7.8 Reusing history $\mathcal{H}$ for a new goal.

Once a goal is selected, we compute the L2 distance between all vectors (c, r_x, r_y) of the history and (c_{goal}, g_x, g_y), where g_x and g_y are the (x,y) coordinate of the goal and c_{goal} is a constant equal to 0.065 in this work. These L2 distances are used to select a point in the history reaching a position close to the goal while mitigating the risk of collapsing.

In addition to these L2 distances for the selection of potential candidates for a new goal, we also filter out the points in the history having $c > 0.11$ allowing to remove the potential collapsing ones even though they might be close to the goal. We also take into account this collapsing proxy measure as collapsing parameters are hard to recover from through gradient descent.

The candidate parameter for a goal is therefore the point in the history which has $c \leq 0.11$ and which minimize the L2 distance presented above.

S7.9 IMGEP search Hyperparameters

- Number of IMGEP steps : 120
- History initialization : 40 trials of random parameters.
- In 4 out of 5 IMGEP step, we mutate the candidate parameter before gradient descent.
- Number of gradient steps : 125 when no mutation beforehand (1 out of 5 IMGEP steps) , 15 when mutation beforehand.
- Rollout length : 50 timesteps
- Grid size : 256x256
- Number of obstacle during the search: 8
- Initialization position on the 256x256 grid: [36:76,105:145]

S8 Basic obstacles tests and generalization tests

Note that the tests we provide are proxy measure of spatial locality/stability. It is for example impossible to test for infinite time stability in finite time budget. Our stability tests are based on previous work on Lenia (*40*).

S8.1 Empirical stable soliton test

We describe here the stable soliton test used in the paper:

We first apply a prefilter to the obtained parameters by running a rollout of 500 steps with the obtained parameters. From this rollout, we measure if the mass at the last timestep was strictly above 0 (not dead) and below 6400 (explosion). The number are arbitrary and relatively "loose" so that we reject nearly no "false positive". This prefilter allows to throw out obvious non interesting parameters to reduce the computational cost of testing all obtained parameters – especially for the random search method where many of them are not interesting.

We then do rollout of 2000 timesteps for the empirical soliton and moving test. The rollout is long (especially relative to the 50 timesteps of the search) in order to probe for long term stability. We compute some stats, from the rollout observations, which are used for the empirical soliton test (and moving test) of the parameters inspired by (*40*).

The empirical soliton test consist of :

- Measuring if the mass of the learnable channel is > 0 and < 6400 ($\sim$10% of the map) at the last timestep of the rollout as those correspond to collapse and explosion.
- Measuring if the average mass is augmenting or decreasing too much between 2 windows of the rollout. This is a proxy measure for long term instability meaning that a big loss or increase of mass between the 2 windows is most of the time an indicator for long term instability. In this work, we measure the ratio between the average mass during the 0 to 500 window and 1500 to 2000 window. If this ratio is greater than 2, the parameters do

not pass the test. The windows are relatively large to still allow for variation of mass during a rollout and the formation of a pattern in the first window.

- We also want the emerging pattern to be a spatially localized **Soliton** (ie pattern forming a single entity not expanding indefinitely, with a bounded radius). To measure this, we perform a connectivity analysis of the pattern depending on the kernel radius, rejecting patterns where two distinct blobs of mass cannot influence each other (distance between blobs $\geq R * max(r)$).

S8.2 Moving test

To test if a pattern passing the empirical stable soliton test is moving, we measure if the center of mass of the learnable channel moved further than 100 pixels from the initialization position at any point during the 1000 first steps of the rollout.

S8.3 Speed measure

To measure speed of solitons, we use the 2000 timesteps rollout computed in the filter phase and track the average distance travelled by the center of mass of the solitons on sliding overlapping windows of size 25 starting from timestep 150 to timestep 2000. The result is divided by 25 (the size of the sliding window) in order to have a per timestep average distance travelled. We use a sliding window to filter slight back and forth movement of the center of mass (which can even be due to self organization without clear "movement" of the whole). Note that we compute the speed only for pattern passing the soliton test above.

The same is done to measure speed with obstacles but we average on the 50 rollouts with random obstacles computed in the robustness test. The only small modification is that if a soliton does not pass the survival tests above on the rollout (for example its mass reaches 0), we set the speed for this rollout to 0.

S8.4 Basic obstacles tests

We then test the parameters leading to moving soltions by performing 50 rollouts of 2000 timesteps where obstacles are the same as in training i.e. obstacles of radius 10. We place 24 obstacles in the whole grid (compared to only the right part of the grid in training), from which 23 are randomly placed and one being in the trajectory of the moving soliton to be sure that it will encounter at least one obstacle in the rollout. To do this we look at the achieved position of the moving soliton without obstacle at timestep 1000 and put an obstacle here in the test for every rollout. We also remove any obstacle pixel in the initialization area (pixel of the learnable channel > 0 at the initialization) as well as in a radius of 10 pixels (euclidian distance) of the initialization (to let some space for the initialization to develop).

From the observations of the rollout we compute the same statistics and same categories used for the soliton test. To get the robustness measure we then measure the fraction of rollout where the pattern pass the empirical soliton test (despite the perturbations by the obstacles).

S8.5 Generalization tests

Here is a full description of each of the generalization test conducted in the *Generalization* section in the main text. For all the quantitative generalization tests, we used the same robustness test as above except that we do it on 10 random trials instead of 50: we run rollout of 2000 timesteps, then measure if it fulfills the empirical soliton test. The measure of robustness is again measured by the proportion of trials where the pattern pass the empirical soliton test. (hence between 0 and 1).

We also provide a more detailed table of generalization results in Tab.S2 adding also patterns obtained through random search and semi-manual search.

- **Initialization noise**. In this experiment, we add a centered gaussian noise to the pixel of the initialization square A^1. In the first test "init noise rate" we vary the proportion of pixels affected by this gaussian noise, testing proportions [0.2,0.4,0.6,0.8,1.], and keep

the variance fixed to 1. In the "init noise std" test, we apply the noise to all pixels of the initialization but vary the variance of the gaussian in [0.5,1.5,2.5,3.5,4.5].

- **Obstacles** In all of these test we also remove obstacles pixel from the initialization square and in a radius of 10 pixels (euclidian distance) around it.
  - **Obstacle radius** In this test, we vary the radius of the obstacles in [4,7,10,13,16]. The number of obstacles varies according to the radius of obstacles to keep the same ratio of obstacle pixels with the default one which is 24 obstacles of radius 10. The formula is Number obstacles $= 24 \times (10/var)^2$.
  - **Obstacles number** In this test, we vary the obstacle number keeping the radius fixed to the default one (radius=10). We try obstacle number= [24, 30,36,42 ,48] .
  - **Obstacle speed**. In this test, we change the dynamic of the obstacle channel so that obstacle move at a certain speed as detailed in S6.2. For a speed of 1, the obstacle channel is shifted of 1 on the left at every timestep, for a speed of 0.5, the obstacle channel is shifted of 1 every 2 timesteps. We tested obstacle speed of [1/3,1/2,1,2,3]. In this test we put 24 obstacles of radius 10.
- **Scale** In this test, we vary the scale of patterns by changing their kernel size multiplying the parameter R of the simulation by the factor. A smaller (resp bigger) size of kernel means that the convolution will cover a smaller (resp bigger) neighbourhood. We also change the initialization size by a factor α to match the scale. To do this, we use a downscaling (or upscaling) of the initialization 40×40 square with bilinear interpolation. We test both smaller sizes : 0.15,0.65 , as well as bigger sizes: 1.15,1.65,2.15.
- **Update**. In this tests, we perturb the update (what is added to the current state) from step 0 until step 1900. We let the step from 1900 to 2000 free of update perturbation to allow the rule to recover until step 2000 for the statistics computation.

- **Update mask** In this test, for a value of update mask $p < 1$, every pixel has a probability p of being updated while the rest of the pixels will keep the same value. This does not apply to the update applied by the obstacles. For a value $1 < p < 2$, each pixel is updated one time using the update rule normally (sensing and add of growth) giving a new state and then each pixel is updated again from this new state with a $p - 1$ probability (the sensing on the potential second random update is done by sensing the new state). We test the update mask rate in [0.2,0.6,1.,1.4,1.8].

- **update noise std** In this test, we add noise to the update of the learnable channel before the clipping as such :

$$A_l^{t+1} = A_l^t + \frac{1}{T}\left(G(K * A^t) + \mathcal{N}(0_{256\times256}, \sigma\mathbb{I}_{256\times256})\right)$$

  where $\mathcal{N}(\mu, \Sigma)$ is a gaussian vector of mean μ and variance Σ. We vary σ in $[0.5, 1.5, 2.5, 3.5, 4.5]$

- **Update noise rate**. We add noise to the update of the learnable channel before clipping. Every pixel has a probability $p \in [0.2, 0.4, 0.6, 0.8, 1.]$ to have a gaussian noise of mean 0 and variance 1.

• **Morphological computation/ Hand damage**. In this test, we allow an exterior experimenter to pause the simulation and put pixels of the learnable channel to 0. After the damage, we then let the simulation unroll as usual starting from the damaged state $A_l^{damaged}$.

• **Interactions (Multi agents setting)**. We allow to put several initialization square in the learnable channel. As the update rule apply to all the grid the same way, if a couple (initialization square, update rule) already led to a soliton in the case of a single initialization square then several of them that are not interfering (further enough so that the convolution of a pixel of one does not contains pixels of the other) will lead to several solitons.

- **Custom obstacles**. We allow an experimenter to freely draw obstacle in the grid. This allows to have obstacles with shapes not seen during training.
- **Custom init states** In this test, we replace the initialization of the pattern (that was optimized) by simple arbitrary shape such as disk with a gradient (the gradient being to have an asymmetry for movement), disk of large size etc. The web demo at http://developmentalsystems.org/sensorimotor-lenia-companion also allows to load any image as initialization of the system.
- **External control** This experiment consists in adding a new channel (a new type of cell) to the system which we want to act as an attractive element. We conducted a semi handmade search in order to search for a rule, sensing in the attractive channel and updating the learnable channel, leading to this attractive behavior.

  Note that this attractive element should attract but not disturb too much the matter as we don't want the attractive matter to be able to destroy the sensorimotor agent dynamics.

  In fact, we first searched for a rule tuned for one sensorimotor agent found with the IMGEP search (ie one parameter point (A_l, θ_l)). By doing so, the rule is adapted to the dynamic of this specific agent (for example different sensorimotor agents might have different range for pixel value or growth etc).

  The search for a rule (tuned for a specific sensorimotor agent) is semi handmade. We first preselect some rule parameters from a set of random rules. The preselection is done by moving a circle of attractive mass along a predefined straight trajectory in an environment with a moving sensorimotor agent. We then look if the attractive mass and the agent overlaps at the last timestep which should mean that the agent followed this attractive mass. An experimenter then select by hand the rules that lead to attraction of mass without too much perturbation by controlling the mass of attractive matter in a real time simulation with the moving sensorimotor agent.

  After searching for a rule for a specific sensorimotor agent, we then tested it on some

other agents obtained with IMGEP. Some agents (some parameters (A_f, θ_f)) are more prone to work with it (meaning attraction while not affecting the stability too much) while it destroy the stability of others. The reported qualitative results on this test are performed on sensorimotor agents where the rule leads to stable attraction.

S9 Comparison baselines

S9.1 Random search details

We use uniform sampling of parameters with the ranges given in S6.3.

The initialization 40x40 square is randomly sampled with each of the pixel constituting it being independently sampled following a uniform distribution between 0 and 1.

S9.2 "Handmade" patterns (from original Lenia paper)

The parameters from this dataset are the one from the original Lenia paper (*30, 31*) (following these links: https://github.com/Chakazul/Lenia/tree/master/Python/found,
and https://github.com/Chakazul/Lenia/blob/master/Python/old/animals.json). Contrary to the rest of the paper we use the classic parameterization of Lenia for the learnable channel. We filter out those that have more than one channel or an initialization that has a side bigger than 256. We then apply the pre-filter and filter as explained in section S8 .1. We provide the resulting parameters in the data folder of https://zenodo.org/records/10211741.

In the handmade search from the original Lenia papers, self-organizing patterns were discovered by basic evolutionary algorithms, through one of these routes: (1) random parameter values and initial patterns; (2) start from an existing moving pattern and mutate the parameter values; (3) manual editing of the initial pattern.

S10 Movie legends

You can find all movies on this companion website https://developmentalsystems.org/sensorimotor-lenia-companion/.

- **Movie S1: Sensorimotor agents** Different sensorimotor agents (yellow) emerging from rules obtained by the IMGEP. The solitons display sensorimotor capabilities: they are robust and react to perturbations by the obstacles (blue). The righmost video shows the system with a different colormap (fixed obstacle channel in black) to highlight the differences in activity in the sensorimotor agent as a response to perturbation.

- **Movie S2: Random search** Each 100 squares are random parameters trials (each 1 channel and 10 rules so 1̃30 parameters for all the rules of a square). We observe that a lot of random search trials lead to death or explosion of the mass. Very little lead to stable spatially localized pattern and even less to moving ones.

- **Movie S3 Orbium, moving soliton from the original lenia papers, fragile to external perturbations** S3.a: Orbium: the equivalent of the glider in Lenia (from the original lenia paper), an example of moving soliton. S3.b and S3.c videos: collision between several orbium leading to death/explosion. This shows the fragility of the orbium to external perturbations.

- **Movie S4 Orbium perturbed by obstacles** Orbium, equivalent of the glider in Lenia (from the original lenia paper), dies from perturbations by obstacles.

- **Movie S5: Solitons obtained by each method** 100 Patterns passing our stable soliton tests obtained by each method: random search(S5.a),IMGEP (S5.b), handmade search ((S5.c)from Lenia original papers). A lot of IMGEP obtained solitons are moving solitons with high speed while a lot of solitons obtained by random search are static.

- **Movie S6: Moving obstacle test on solitons obtained by each method** 100 solitons obtained by random search(S6.a),IMGEP(S6.b), handmade search ((S6.c) from Lenia original papers). We observe that the proportion of solitons with robustness to moving obstacles is much higher in the solitons obtained by IMGEP than the ones obtained by random search and handmade search.

- **Movie S7: Illustration of the quantitative generalization tests performed** See companion website https://developmentalsystems.org/sensorimotor-lenia-companion/. Videos of quantitative tests for sensorimotor agents obtained by IMGEP. We display only a subset of the value tested for every quantitative test.

- **Movie S8: Out of distribution obstacles: Different shapes** Test of a sensorimotor agent obtained by IMGEP on obstacles that were not seen during training.

- **Movie S9: Out of distribution obstacles: maze.** Test of a moving sensorimotor agent to maze like obstacles.

- **Movie S10: Out of distribution obstacles: Bullet like obstacles** Test of a moving sensorimotor agent to bullet like environment: fast small moving obstacles.

- **Movie S11: Individuality preservation** Example of sensorimotor agents obtained by IMGEP colliding while keeping their individuality, they don't merge or collapse from the collision.

- **Movie S12: Reproduction** For some sensorimotor agents, under specific conditions, the collision of 2 agents can lead to the self-organization of a 3rd agent (each with its own individuality).

- **Movie S13: Attraction** Example of sensorimotor agents attracting each other while still maintaining their own individuality.

- **Movie S14: Asynchronous update** Testing a sensorimotor agent with asynchronous updates. Each cell is updated with a certain probability at each step leading to cells being asynchronously updated.

- **Movie S15: Scaling the agents down** The moving agents size is reduced. The scaled down agents still seem to behave similarly (same shape and have sensorimotor capabilities) to the normal size one while being composed of less cells.

- **Movie S16: Morphological computation** We pause the simulation and remove some cells of a moving agent. As a response to this alteration of the structure, the moving agent changes direction, regrow itself and moves away. This video isolates the fact that the macro agent senses perturbations of its structure and respond to it by a morphological growth.

- **Movie S17: External control.** We introduce an attractive element in another channel (in Cyan). We learned the rule that control the way this external element channel influences the learnable channel(Yellow) and display the resulting behavior here. The moving agent is effectively attracted to this introduce component. By controlling the external element we can control live the direction of the moving agent.

- **Movie S18: Robustness to initialization** Testing the robustness of the learned rule to emerge an agent from different initial patterns. We replace the learned initial pattern by : S18.left a disk with a gradient; S18.middle a large disk (much larger than an agent); S18.right top a disk with gradient of another size, bottom a disk without gradient. Some initialization lead to the robust emergence of one or several agents while some lead to the collapse of the pattern.

- **Movie S19: Examples of solitons considered non moving by our moving test**

- **Movie S20: Damaged agents preserving sensorimotor capabilities**

REFERENCES AND NOTES

1. E. A. Di Paolo, Process and individuation: The development of sensorimotor agency. *Hum. Dev.* **63**, 202–226 (2019).

2. E. Di Paolo, Does agency come in levels? *Spontaneous Generations* **11**, 10.4245/spongen.v11i1.22835 (2023).

3. S. E. Sultan, A. P. Moczek, D. Walsh, Bridging the explanatory gaps: What can we learn from a biological agency perspective? *BioEssays* **44**, e2100185 (2022).

4. E. A. Di Paolo, Autopoiesis, adaptivity, teleology, agency. *Phenom. Cogn. Sci.* **4**, 429–452 (2005).

5. X. E. Barandiaran, E. Di Paolo, M. Rohde, Defining agency: Individuality, normativity, asymmetry, and spatio-temporality in action. *Adapt. Behav.* **17**, 367–386 (2009).

6. A. Moreno, A. Etxeberria, Agency in natural and artificial systems. *Artif. Life* **11**, 161–175 (2005).

7. M. Levin, Technological approach to mind everywhere: An experimentally-grounded framework for understanding diverse bodies and minds. *Front. Syst. Neurosci.* **16**, 768201 (2022).

8. M. Etcheverry, C. Moulin-Frier, P.-Y. Oudeyer, M. Levin, AI-driven automated discovery tools reveal diverse behavioral competencies of biological networks. *eLife* **13**, RP92683 (2025).

9. C. G. Langton, *Artificial Life: An Overview* (MIT Press, 1995).

10. R. D. Beer, The cognitive domain of a glider in the game of life. *Artif. Life* **20**, 183–206 (2014).

11. A. Moreno, On minimal autonomous agency: Natural and artificial. *Complex Syst.* **27**, 289–313 (2018).

12. M. Biehl, N. Virgo, “Interpreting systems as solving POMPDs: A step towards a formal understanding of agency” in *International Workshop on Active Inference* (Springer, 2022), pp. 16–31.

13. D. Abel, A. Barreto, M. Bowling, W. Dabney, S. Dong, S. Hansen, A. Harutyunyan, K. Khetarpal, C. Lyle, R. Pascanu, G. Piliouras, D. Precup, J. Richens, M. Rowland, T. Schaul, S. Singh, Agency is frame-dependent. arXiv:2502.04403 (2025).

14. S. J. Russell, P. Norvig, *Artificial Intelligence: A Modern Approach* (Pearson, 2016).

15. P. Lyon, F. Keijzer, D. Arendt, M. Levin, Reframing cognition: Getting down to biological basics. *Philos. Trans. R. Soc. B Biol. Sci.* **376**, 20190750 (2021).

16. R. Wang, J. Lehman, J. Clune, K. O. Stanley, Paired open-ended trailblazer (POET): Endlessly generating increasingly complex and diverse learning environments and their solutions. *CoRR*, abs/1901.01753 (2019).

17. I. Akkaya, M. Andrychowicz, M. Chociej, M. Litwin, B. McGrew, A. Petron, A. Paino, M. Plappert, G. Powell, R. Ribas, J. Schneider, N. Tezak, J. Tworek, P. Welinder, L. Weng, Q. Yuan, W. Zaremba, L. Zhang, Solving rubik's cube with a robot hand. arXiv:1910.07113 (2019).

18. B. Baker, I. Kanitscheider, T. Markov, Y. Wu, G. Powell, B. McGrew, I. Mordatch, Emergent tool use from multi-agent autocurricula. arXiv:1909.07528 (2019).

19. O. E. L. Team, A. Stooke, A. Mahajan, C. Barros, C. Deck, J. Bauer, J. Sygnowski, M. Trebacz, M. Jaderberg, M. Mathieu, N. M. Aleese, N. Bradley-Schmieg, N. Wong, N. Porcel, R. Raileanu, S. Hughes-Fitt, V. Dalibard, W. M. Czarnecki, Open-ended learning leads to generally capable agents. arXiv:2107.12808 (2021).

20. R. Pfeifer, J. Bongard, *How the Body Shapes the Way We Think: A New View of Intelligence* (MIT Press, 2006).

21. T. Froese, T. Ziemke, Enactive artificial intelligence: Investigating the systemic organization of life and mind. *Artif. Intel.* **173**, 466–500 (2009).

22. F. Varela, H. Maturana, R. Uribe, Autopoiesis: The organization of living systems, its characterization and a model. *Biosystems* **5**, 187–196 (1974).

23. B. McMullin, Thirty years of computational autopoiesis: A review. *Artif. Life* **10**, 277–295 (2004).

24. R. D. Beer, Autopoiesis and cognition in the game of life. *Artif. Life* **10**, 309–326 (2004).

25. E. Agmon, A. J. Gates, R. D. Beer, "Ontogeny and adaptivity in a model protocell" in *Artificial Life Conference Proceedings 13* (MIT Press, 2015), pp. 216–223.

26. D. Krakauer, N. Bertschinger, E. Olbrich, J. Flack, N. Ay, The information theory of individuality. *Theory Biosci.* **139**, 209–223 (2020).

27. M. Biehl, T. Ikegami, D. Polani, "Towards information based spatiotemporal patterns as a foundation for agent representation in dynamical systems" in *Proceedings of the Artificial Life Conference 2016* (MIT Press, 2016), pp. 722–729.

28. R. D. Beer, Bittorio revisited: Structural coupling in the game of life. *Adapt. Behav.* **28**, 197–212 (2020).

29. A. Cika, E. Cohen, G. Kruszewski, L. Seet, P. Steinmann, W. Yin, "Resilient life: An exploration of perturbed autopoietic patterns in Conway's Game of Life" in *ALIFE 2020: The 2020 Conference on Artificial Life* (The International Society for Artificial Life, 2020), pp. 656–664.

30. B. W.-C. Chan, Lenia: Biology of artificial life. *Complex Syst.* **28**, 251–286 (2019).

31. B. W.-C. Chan, "Lenia and expanded universe", in *The 2020 Conference on Artificial Life* (MIT Press, 2020), pp. 221–229.

32. M. Etcheverry, "Curiosity-driven AI for science: Automated discovery of self-organized structures," thesis, Université de Bordeaux (2023).

33. I. Yevenko, "Classifying the fractal parameter space of the Lenia Orbium" in *ALIFE 2024: Proceedings of the 2024 Artificial Life Conference* (The International Society for Artificial Life, 2024), pp. 14.

34. A. Baranes, P.-Y. Oudeyer, Active learning of inverse models with intrinsically motivated goal exploration in robots. *Robot. Autonom. Syst.* **61**, 49–73 (2013).

35. S. Forestier, R. Portelas, Y. Mollard, P.-Y. Oudeyer, Intrinsically motivated goal exploration processes with automatic curriculum learning. *J. Mach. Learn. Res.* **23**, 1–41 (2022).

36. C. Colas, P. Fournier, M. Chetouani, O. Sigaud, P.-Y. Oudeyer, "Curious: Intrinsically motivated modular multi-goal reinforcement learning" in *International Conference on Machine Learning* (PMLR, 2019), pp. 1331–1340.

37. C. Colas, T. Karch, N. Lair, J.-M. Dussoux, C. Moulin-Frier, P. Dominey, P.-Y. Oudeyer, Language as a cognitive tool to imagine goals in curiosity driven exploration. *Adv. Neural Inf. Process. Syst.* **33**, 3761–3774 (2020).

38. J. Grizou, L. J. Points, A. Sharma, L. Cronin, A curious formulation robot enables the discovery of a novel protocell behavior. *Sci. Adv.* **6**, eaay4237 (2020).

39. M. J. Falk, F. D. Roach, W. Gilpin, A. Murugan, Curiosity-driven search for novel non-equilibrium behaviors. *Phys. Rev. Res.* **6**, 033052 (2024).

40. C. Reinke, M. Etcheverry, P.-Y. Oudeyer, Intrinsically motivated discovery of diverse patterns in self-organizing systems. arXiv:1908.06663 (2020).

41. M. Etcheverry, C. Moulin-Frier, P.-Y. Oudeyer, "Hierarchically organized latent modules for exploratory search in morphogenetic systems" in *Advances in Neural Information Processing Systems*, vol. 33, H. Larochelle, M. Ranzato, R. Hadsell, M. F. Balcan, H. Lin, Eds. (Curran Associates, Inc., 2020), pp. 4846–4859.

42. H. Kitano, Biological robustness. *Nat. Rev. Genet.* **5**, 826–837 (2004).

43. S. Wolfram, Universality and complexity in cellular automata. *Phys. D Nonlinear Phenom.* **10**, 1–35 (1984).

44. J. Gottlieb, P.-Y. Oudeyer, Towards a neuroscience of active sampling and curiosity. *Nat. Rev. Neurosci.* **19**, 758–770 (2018).

45. A. Mordvintsev, E. Randazzo, E. Niklasson, M. Levin, S. Greydanus, Thread: Differentiable self-organizing systems. *Distill*, 10.23915/distill.00027 (2020).

46. A. Mordvintsev, E. Randazzo, E. Niklasson, M. Levin, Growing neural cellular automata. *Distill*, 10.23915/distill.00023 (2020).

47. E. Niklasson, A. Mordvintsev, E. Randazzo, M. Levin, Self-organising textures. *Distill*, 10.23915/distill.00027.003 (2021).

48. E. Randazzo, A. Mordvintsev, E. Niklasson, M. Levin, S. Greydanus, Self-classifying mnist digits. *Distill*, 10.23915/distill.00027.002 (2020).

49. A. Variengien, S. Pontes-Filho, T. E. Glover, S. Nichele, Towards self-organized control: Using neural cellular automata to robustly control a cart-pole agent. arXiv:2106.15240 (2021).

50. H. R. Maturana, F. J. Varela, *Autopoiesis and Cognition: The Realization of the Living* (Springer Dordrecht, 1980).

51. L. N. Vandenberg, D. S. Adams, M. Levin, Normalized shape and location of perturbed craniofacial structures in the *Xenopus* tadpole reveal an innate ability to achieve correct morphology. *Dev. Dyn.* **241**, 863–878 (2012).

52. G. Fankhauser, Maintenance of normal structure in heteroploid salamander larvae, through compensation of changes in cell size by adjustment of cell number and cell shape. *J. Exp. Zool.* **100**, 445–455 (1945).

53. J. Stelling, U. Sauer, Z. Szallasi, F. J. Doyle III, J. Doyle, Robustness of cellular functions. *Cell* **118**, 675–685 (2004).

54. M. Levin, The computational boundary of a "self": Developmental bioelectricity drives multicellularity and scale-free cognition. *Front. Psychol.* **10**, 2688 (2019).

55. W. Li, X. Wu, H. Qin, Z. Zhao, H. Liu, Light-driven and light-guided microswimmers. *Adv. Funct. Mater.* **26**, 3164–3171 (2016).

56. R. S. Sutton, A. G. Barto, *Reinforcement Learning: An Introduction,* vol. 1 (MIT Press, 1998).

57. T. Bansal, J. Pachocki, S. Sidor, I. Sutskever, I. Mordatch, Emergent complexity via multi-agent competition. *CoRR*, abs/1710.03748 (2017).

58. R. Solé, C. P. Kempes, B. Corominas-Murtra, M. De Domenico, A. Kolchinsky, M. Lachmann, E. Libby, S. Saavedra, E. Smith, D. Wolpert, Fundamental constraints to the logic of living systems. *Interface Focus* **14**, 20240010 (2024).

59. C. M. E. Kriebisch, O. Bantysh, L. B. Pellejero, A. Belluati, E. Bertosin, K. Dai, M. de Roy, H. Fu, N. Galvanetto, J. M. Gibbs, S. S. Gomez, G. Granatelli, A. Griffo, M. Guix, C. O. Gurdap, J. Harth-Kitzerow, I. S. Haugerud, G. Häfner, P. Jaiswal, S. Javed, A. Karimi, S. Kato, B. A. K. Kriebisch, S. Laha, P.-W. Lee, W. P. Lipinski, T. Matreux, T. C. T. Michaels, E. Poppleton, A. Ruf, A. D. Slootbeek, I. B. A. Smokers, H. Soria-Carrera, A. Sorrenti, M. Stasi, A. Stevenson, A. Thatte, M. Tran, M. H. I. van Haren, H. D. Vuijk, S. F. J. Wickham, P. Zambrano, K. P. Adamala, K. Alim, E. S. Andersen, C. Bonfio, D. Braun, E. Frey, U. Gerland, W. T. S. Huck, F. Jülicher, N. Laohakunakorn, L. Mahadavan, S. Otto, J. Saenz, P. Schwille, K. Göpfrich, C. A. Weber, J. Boekhoven, A roadmap toward the synthesis of life. *Chem* **11**, 102399 (2025).

60. S. Bartlett, M. L. Wong, Defining lyfe in the universe: From three privileged functions to four pillars. *Life* **10**, 42 (2020).

61. B. Baker, I. Kanitscheider, T. Markov, Y. Wu, G. Powell, B. McGrew, I. Mordatch, Emergent tool use from multi-agent autocurricula. arXiv:1909.07528 (2020).

62. E. Plantec, G. Hamon, M. Etcheverry, P.-Y. Oudeyer, C. Moulin-Frier, B. W.-C. Chan,Flow-lenia: Towards open-ended evolution in cellular automata through mass conservation and parameter localization, ALIFE 2023: Ghost in the Machine, *Proceedings of the 2023 Artificial Life Conference*, 24 to 28 July 2023 online (MIT Press, 2023), pp. 131.

63. K. O. Stanley, J. Lehman, L. Soros, "Open-endedness: The last grand challenge you've never heard of," *O'Reilly Online* (2017); https://www.oreilly.com/radar/open-endedness-the-last-grand-challenge-youve-never-heard-of/.

64. B. W.-C. Chan, Towards large-scale simulations of open-ended evolution in continuous cellular automata. arXiv:2304.05639 (2023).

65. G. Pezzulo, M. Levin, Re-membering the body: Applications of computational neuroscience to the top-down control of regeneration of limbs and other complex organs. *Integr. Biol.* **7**, 1487–1517 (2015).

66. G. Pezzulo, M. Levin, Top-down models in biology: Explanation and control of complex living systems above the molecular level. *J. R. Soc. Interf.* **13**, 20160555 (2016).

67. R. Nakano, J. Hilton, S. Balaji, J. Wu, L. Ouyang, C. Kim, C. Hesse, S. Jain, V. Kosaraju, W. Saunders, X. Jiang, K. Cobbe, T. Eloundou, G. Krueger, K. Button, M. Knight, B. Chess, J. Schulman, Webgpt: Browser-assisted question-answering with human feedback. arXiv:2112.09332 (2021).

68. T. Schick, J. Dwivedi-Yu, R. Dessí, R. Raileanu, M. Lomeli, L. Zettlemoyer, N. Cancedda, T. Scialom, Toolformer: Language models can teach themselves to use tools. arXiv:2302.04761 (2023).

69. S. Kriegman, D. Blackiston, M. Levin, J. Bongard, A scalable pipeline for designing reconfigurable organisms. *Proc. Natl. Acad. Sci. U.S.A.* **117**, 1853–1859 (2020).

70. M. R. Ebrahimkhani, M. Levin, Synthetic living machines: A new window on life. *Iscience* **24**, 102505 (2021).

71. R. Portelas, C. Colas, L. Weng, K. Hofmann, P.-Y. Oudeyer, Automatic curriculum learning for deep RL: A short survey. arXiv:2003.04664 (2020).

72. W. Gilpin, Cellular automata as convolutional neural networks. *Phys. Rev. E* **100**, 032402 (2019).

73. H. S. Kotian, A. Z. Abdulla, K. N. Hithysini, S. Harkar, S. Joge, A. Mishra, V. Singh, M. M. Varma, Active modulation of surfactant-driven flow instabilities by swarming bacteria. *Phys. Rev. E* **101**, 012407 (2020).
